# An Integrative Approach to Hazardous Effects Caused by Pharmaceutical Contaminants on Aquatic Effluents

**DOI:** 10.3390/molecules30173483

**Published:** 2025-08-25

**Authors:** Irina Meghea, Daniela Simina Stefan, Florina Ioniţă, Mihai Lesnic, Ana-Maria Manea-Saghin

**Affiliations:** 1 Department of Mathematical Methods and Models, Faculty of Applied Sciences, National University of Science and Technology POLITEHNICA Bucharest, 313 Splaiul Independentei, 060042 Bucharest, Romania; irina.meghea@upb.ro; 2 Research Centre for Environmental Protection and Eco-Friendly Technologies, National University of Science and Technology POLITEHNICA Bucharest, 1 Polizu Street, 011061 Bucharest, Romania; mlesnic@yahoo.com; 3 Department of Inorganic Technology, Faculty of Chemical Engineering and Biotechnologies, National University of Science and Technology POLITEHNICA Bucharest, 1 Polizu Street, 011061 Bucharest, Romania; daniela.stefan@upb.ro (D.S.S.); florina.lungu@stud.chimie.upb.ro (F.I.)

**Keywords:** pharmaceutical contaminants, wastewater cleaning, pollution abatement, rehabilitation of treatment stations, hazardous environmental effects, admitted limits, remediation strategies

## Abstract

This study presents a general overview of the important problem of pharmaceutical pollutants, aiming to draw attention to the global danger they represent and the need for concrete solutions for their remediation. Here, we summarize the available advanced knowledge on the occurrence and fate of pharmaceutical residues in the environment, particularly in water effluents, since they need a special approach when one takes into account the increasing consumption of medicines by both humans and animals, that might be discharged in aqueous systems and bio-accumulated in aquatic flora and fauna. This review details the presence of pharmaceutical wastes in water sources; their trajectories from production to consumption and release in household taps; their concentrations in natural water; methods for monitoring them; their risks; and their worldwide impacts. Adequate methods and advanced removal techniques for selected contaminants or classes of pharmaceutical compounds are discussed, together with their remediation potential and strategies. Local and global limiting proposals for these types of contaminants and concrete solutions for their remediation are recommended.

## 1. Introduction

Even though the wastes generated by pharmaceutical products (PhPs) exhibit some characteristic properties similar to those of persistent organic pollutants (POPs), being non-biodegradable, bio-accumulative, or even toxic, they are not included in the catalogue of dangerous compounds nominated according to the Stockholm Convention: aldrin, chlordane, DDT (dichlorodiphenyltrichloroethane), dieldrin, dioxins, heptachlor, endrin, furans, hexachlorobenzene, mirex, polychlorinated biphenyls (PCBs), toxaphene, octabrom diphenyl ether, hexachlorocyclohexane (HCH), lindane, hexabromobiphenyl, and perfluoro octane sulphonate.

In recent years, pharmaceutical compounds have been found within the water cycle, namely in surface and ground water, waste water, and even in drinkable water at the levels of micrograms per litre [1]. In the last three decades, pharmaceutical residues have been detected in lakes, water flows, estuaries, and rivers; they have also been detected in influents, effluents, and sludge of treatment stations on all continents, even in polar regions, including Antarctica [2], where some substances affecting endocrine glands, along with some antimicrobial substances and synthetic hormones, have been identified.

Most pharmaceutical substances are not very persistent, but their continuous addition in small amounts from several sources could cause significant hazardous effects.

Pharmaceutical products include a large number of substances. On the European Union market, more than 3000 frequently used PhPs have been registered [3]. As we mentioned above, pharmaceutical contaminants (PhCs) are not very stable in the environment, but by continuously adding them, along with other pollutants, they could have a significant impact; this is why, in the specialist literature, they are referred to as “pseudo-persistent” [2,4,5].

Pharmaceutical compounds cannot be considered a homogeneous group of compounds, such as CFCs (chlorofluorocarbons), PCBs, or PAHs (polyaromatic hydrocarbons), nor do they have similar biochemical characteristics. Most PhPs are substituted compounds, and they have a specific role in the organism [3]. As a result, most PhCs are distinguished by the following properties [6]: polarity, polymorphism, lipophilicity, enantiomorphism, and various ionization degrees.

Their molar mass is higher than those of other contaminants (>500 Da). Moreover, PhCs have the ability to persist in the natural environment; thus, they are concentrated in diverse life forms. These substances can be distributed in living bodies and are thus metabolized by changing their chemical composition [2,7,8].

At present, a periodic inventory of PhPs should be made in order to take necessary measures to counteract their presence in the environment. However, the control of PhPs is in an incipient stage. The adverse effects of pharmaceuticals on ecosystems and human health have only recently been discovered, and the identification of these pollutants in aquatic systems requires significant financial efforts and specially qualified staff.

This paper is organized into the following three sections: Firstly, the appearance, sources, trajectories, and fates of PhCs are discussed, and their concentrations, pharmaceutical production and consumption routes, monitoring approaches, statistics, and impacts are analysed. The next section discusses the removal of PhCs from water systems, reviewing the adequate methods and cleaning technologies that have been proposed in numerous studies while also highlighting the remediation potential and strategies proposed. The final section emphasizes the feasibility of the proposed depollution solutions, the applicability of remediation strategies, and the urgency with which these recommendations should be implemented.

**Figure 1:**
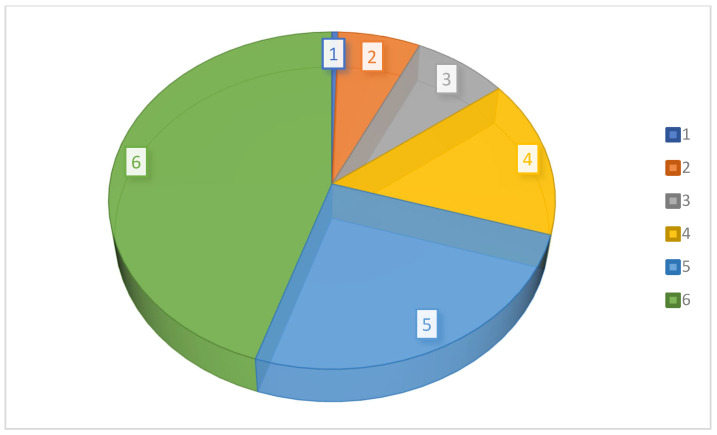
A representation of the number of works cited in this paper across different periods of time: 1—before 2000; 2—from 2001 to 2005; 3—from 2006 to 2010; 4—from 2011 to 2015; 5—from 2016 to 2020; 6—from 2021 to 2025.

The works taken into consideration in this review were collected to substantiate and justify the conclusions drawn and provide a basis for our proposals and recommendations. Given the vastness of the literature in this field, this review cannot, unfortunately, be exhaustive; however, one of our main goals is to develop a database containing, among other things, all major research trends in the field. We reviewed a total of 196 works: 1 was published before 1999, 23 were published between 2000 and 2009, 72 were published between 2010 and 2019, and 101 were published from 2020 onwards. The number of papers in each time period demonstrate the significant increase in interest in this subject in recent years. The time distribution of the number of works in this field is represented in Figure 1.

## 2. Presence of Pharmaceutical Substances in Aquatic Systems

In this section, an exhaustive discussion of the occurrence, sources, fate, trajectories, monitoring, and impact of pharmaceutical wastes is presented. A selective list of some PhPs or classes of PhPs, together with their detected concentrations in a series of countries and ecosystems, is presented in Table A1 (Appendix A.1).

### 2.1. Occurrence, Pollution Sources, and Existing Techniques for Pharmaceuticals Removal

Many comprehensive reviews have recently become available on the occurrence, sources, and mitigation of pharmaceutical contaminants in aquatic systems.

As a consequence of the increasing amounts of water consumed by populations, pharmaceutical industries, and hospitals, huge volumes of effluents containing pharmaceutical pollutants are generated [9]. Detection of PhCs in aquatic systems has been reported from early 1970s [10]. Even though there is consistent progress in the sustainable management of these contaminants, efforts to reduce this pollution are advancing on a rather small scale. The world’s biggest rivers, including the Danube, Tiber, Seine, Thames, and Mekong Rivers, are highly contaminated [11,12].

Moreover, the contaminants in Mediterranean rivers have been summarized by Kock-Schulmeyer et al. [13], while a wide range of PhPs and personal care products in urban and wastewater in Greece have been investigated by Papageorgiou et al. [14].

A worldwide picture of contaminant distribution can be obtained through a comparative analysis of reports from various countries. Thus, Azzouz et al. [15] reported on the determination of selected endocrine disruptive chemicals found in fish and seafood from European and North African markets. Most studies on this topic are coming from researchers in India [11,12,13,14,15,16,17,18] and China [19,20,21,22,23,24]. There are also topical reports from researchers in other countries: Tunisia [25,26], Pakistan [27], Ghana [28], Brazil [29,30], Morocco [31,32,33], Portugal [34], Sri-Lanka [35], Kenya [36], South Africa [37], etc.

Special attention regarding fate and occurrence processes in water treatment has been given to nonsteroidal anti-inflammatory drugs (NSAIDs) and antibiotics [38], which are increasingly used as a result of the recent increasing need to treat various viruses, particularly coronavirus (COVID-19). These drugs are responsible for affecting the endocrine and immune systems in humans. However, such contaminants can be introduced into water-receiving bodies either directly, through animal and human excretion, or indirectly, through unregulated disposal and other anthropogenic activities, which explains their presence in small quantities in aquatic systems. Even though they are present in small amounts, these contaminants are persistent or only partially destroyed during the treatment process and may therefore cause adverse effects on human and animal health due to bioaccumulation.

Other recent reviews on NSAIDs contaminants have been provided in [39,40,41,42].

Huynh et al. [39] showed that many pharmaceuticals are frequently used not only for human therapeutics but also for pet therapies and veterinary practices, thereby increasing the presence of these contaminants in soil, sediments, wastewater, and even in sea water. Among such hazardous pollutants are ibuprofen, ketoprofen, diclofenac, naproxen, and aspirin, which are responsible for endocrine disruption, genotoxicity, organs damage, locomotive disorders, etc. For their removal, the most robust techniques seem to be adsorption using advanced porous carbons and metal–organic composites.

Kumar et al. [41] reviewed emerging contaminants of environmental concern, i.e., synthetic or natural chemicals which are not commonly monitored but which are able to have hazardous effects on human health and the environment, as they can disrupt the physiologies of various receptors. The principal classes of emerging contaminants include pesticides, insecticides, plasticizers, pharmaceutical and personal care products, and fire retardants. Some of these compounds are known as endocrine-disruptive compounds as a result of their adverse effects on the endocrine system. They can be eliminated by membrane technologies. Study results have demonstrated that ultrafiltration, nanofiltration, or reverse osmosis are also efficient techniques for PhC removal. Four NSAID compounds, i.e., naproxen, ibuprofen, diclofenac, and ketoprofen, have been tested in different redox conditions—aerobic, anaerobic, anoxic, and sulphate-reducing conditions—in order to assess their biotic or abiotic degradation in river water/sediment systems [42].

Zhang et al. [43] presented a critical review on the risks and strategies associated with the removal of pyrazolones. These analgesic and anti-inflammatory drugs are largely used for their effectiveness in relieving pain; at the same time, they can pose potential risks, and can be persistent organic pollutants. It is compulsory to develop cost-effective and new environmentally friendly strategies for counteracting the hazardous effects of pyrazolones on human health and the environment. It has been demonstrated that the intermediate products generated during the advanced oxidation treatment of pyrazolones can be more highly toxic than their initial compounds. Biological treatment methods can destroy organic pollutants, and these are promising alternatives for reducing the toxicity of wastewater contaminated with pyrazolones. Innovative technical solutions have been identified through using a bioreactor in the degradation of pyrazolones, combined with other removal techniques that have been proposed to improve their biodegradation.

Lin et al. [44] employed tricarbaldehyde and diaminoguanidine derivatives to develop a novel, leaf-like ionic covalent organic composite for selective NSAID removal. The presence of aromatic, hydroxyl, and guanidinium groups on the materials’ surfaces facilitated the elimination of pharmaceutical contaminants by means of hydrogen bonding, electrostatic, and π → π interactions. It has also been shown that these materials have very good stability and reusability during six adsorption–desorption cycles. These materials are endowed with powerful, selective adsorption capacities for pharmaceutical contaminants, with applications with diclofenac attaining the largest adsorption capacity ever achieved.

At the same time, there are consistent studies directed toward the removal of PhCs by adequate degradation techniques, such as the electro-peroxone process reported by Li et al. [45]. The electro-peroxone procedure combines electrolysis with a conventional ozonation process, while using a carbon–polytetrafluorethylene cathode to electrogenerate hydrogene peroxide from the ozone. In this system, oxydril radicals that are able to oxidize pollutants from a bulk solution are formed. In this way, it was possible for ibuprofen to be more rapidly and completely degraded, as compared with other advanced oxidation processes; thus, the electro-peroxone effluent exhibited much lower toxicity, and is considered to be a promising technology for pharmaceutical contaminant removal.

A review on ibuprofen adsorption on various modified or unmodified porous carbonaceous materials has been presented by Ayati et al. [46]. The following adsorbents were studied in particular: activated carbon, biochar, carbon nanostructured materials, and graphene-based biocomposites.

Removal of NSAIDs, such as ibuprofen, ketoprofen, naproxen, and diclofenac, has been performed using real solutions with high-surface-area nanographene by Al-Khateeb et al. [47]. Advanced characterization techniques revealed the presence of graphene in the form of nanoplatelets, with an average thickness of 5.0 nm. The adsorption experiments showed the high removal efficiency of these contaminants from wastewater.

A powder of waste tire rubber, modified with chitosan, was used by Phasuphan et al. [48] in the removal of ibuprofen, naproxen, and diclofenac from water. The optimal pH for the removal of these three drugs was equal to 6 and the adsorption mechanism was described through pseudo-second order kinetics. The adsorption equilibrium followed the Freundlich isotherm, what is assessed to a heterogeneous surface with different active sites from energy point of view.

A biological process was used by Yu et al. [49] to remove four antibiotics (trimethoprim, sulfadimethoxine, sulfamethazine, and sulfamethoxazole) and four NSAIDs (ibuprofen, ketoprofen, acetaminophen, and naproxen) from water influents. The removal efficiency varied in an inversely proportional manner with contaminant concentration, except for ibuprofen. The quantity, biomass, and reactor size were adequately selected in order to achieve optimal efficiency and continuous removal.

Moreover, a hybrid aerogel, obtained from a thermally activated gelatine–chitosan and amine-functionalized metal–organic derivative, was reported by Kim et al. [50] for the efficient removal of ibuprofen and naproxen. Due to the synergistic advantages of chitosan, gelatine, and the metal–organic compound, doped with zirconium, this new synthetized material exhibited a high adsorption performance on a wide pH range, in addition to a rapid adsorption rate, good thermal stability, and easy recovery. All these properties provide sufficient arguments to demonstrate that this aerogel composite can be successfully used as a good adsorbent for pharmaceutical contaminants from aqueous systems.

A removal mechanism for some anti-inflammatory drugs (aspirin and phenacetin) by an ozone-activated peroxymonosulphate system was proposed by Tan et al. [51]. Ozone, hydroxyl, and sulphate radicals are highly efficient reactive species for the removal of targeted pollutants. A number of five disinfection byproducts were observed from post-chlorine tests, while the toxicity of the intermediates decreased with the increase in pH.

### 2.2. Trajectories of Pharmaceutical Substances from Production, Consumption and Evacuation in Environment Until Household Tap

Pharmaceutical products are manufactured in pharmaceutical industry facilities that are designed to meet the demand for the medicines that are prescribed by doctors. The pharmaceutical industry is often considered a part of the chemical industry. However, the huge diversity and big number of PhPs, their rates of multiplication being evaluated at 6.5 annually, in accordance with OECD [52], have determined a special approach of this sector. The number of pharmaceutical ingredients destined for consumption by the public and in the veterinary domain was approximated to be circa 4000 [53]. About 30–90% of the pharmaceuticals administered to humans and animals are eliminated via urine and faeces [54].

**Figure 2:**
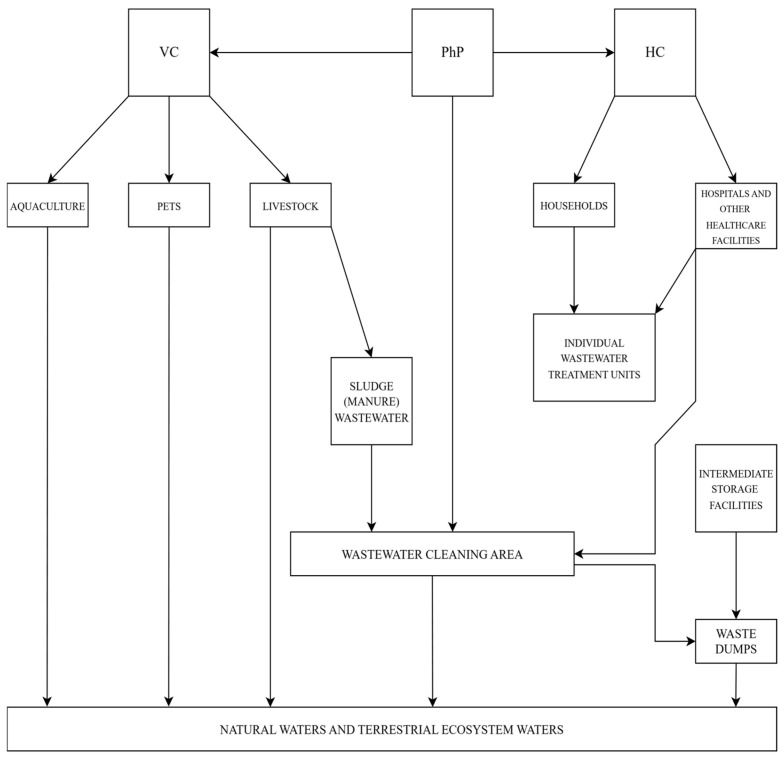
Main discharge routes of pharmaceutical products (PhPs) consumed via human consumption (HC) and veterinary consumption (VC) into the environment.

A scheme of the trajectories followed by PhCs as they reach natural water and other terrestrial ways is presented in Figure 2. From this figure, one can observe that PhPs are directed towards humans or for veterinary use. Human consumption (HC) is partially conducted in households or hospitals and other facilities for healthcare, as well as polyclinics, spa resorts, etc. Veterinary consumption (VC) usually occurs in locations where agricultural activities occur, including aquaculture areas and livestock or pet farms. Both categories of consumption lead to the release of pharmaceutical residues as liquid or solid excretions into natural water (surface or ground). Liquid products are evacuated in natural water directly or via individual or public wastewater treatment plants. Solid products are temporarily deposited and transported to landfills as manure for livestock farms or as fertilizer for agricultural lands. The liquid separated from sludge is discharged to natural water flows, either directly or via wastewater treatment plants. This occurs directly through soil infiltration into groundwater or via discharge into surface water.

**Figure 3:**
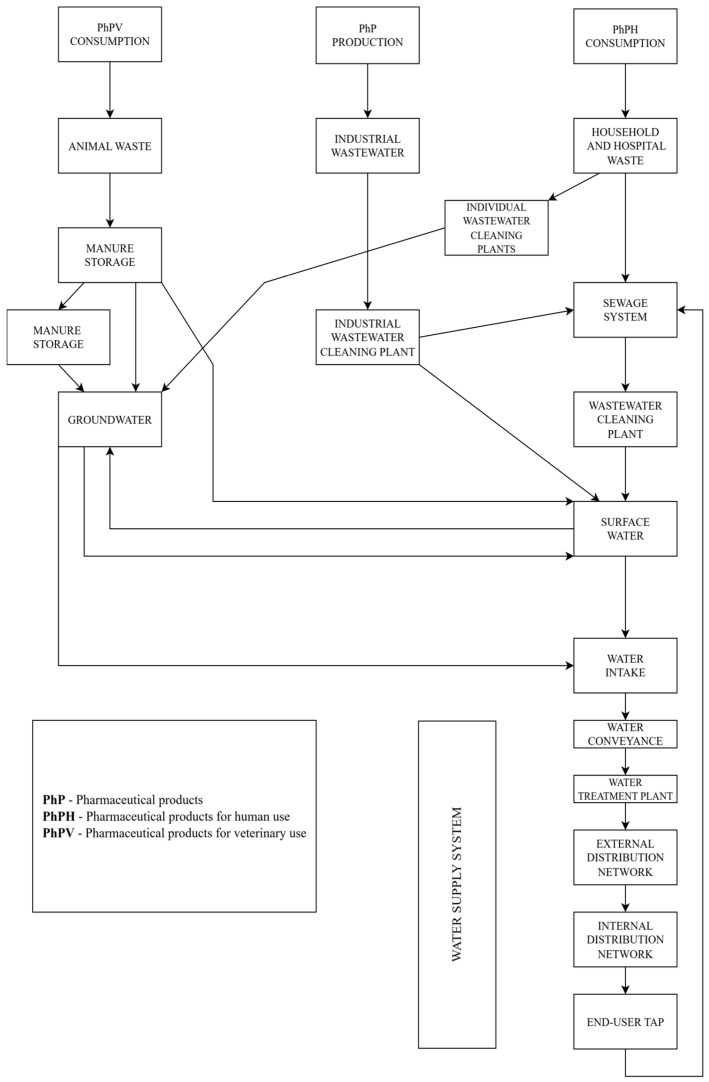
The transport of pharmaceutical pollutants from the producer to the natural water and then to the consumers of drinking water; PhPVs—pharmaceutical products for veterinary use; PhPs—pharmaceutical products; PhPHs—pharmaceutical products for human use.

Figure 3 completes Figure 2, showing the way in which PhCs are transported towards natural water sources and then towards consumers directly or via drinkable water treatment plants. One may observe that there are two types of drug consumers, human (HDC) and animal (ADC), but only one drinking water consumer (DWC).

Over the past 30 years, many countries have raised serious concerns about the presence of PhCs in the environment. Detailed studies conducted in the USA have confirmed the presence of analgesic, estrogenic, and anti-inflammatory drugs in surface water.

An ample review aiming to reveal the presence of PhCs in aquatic systems was provided by Kolpin et al. [55]; through geological investigations conducted during 1999–2000, researchers found more than 50 PhCs in 139 water flows in 30 American States. Most studies have indicated that the persistence of PhCs in surface water is due to human and animal excretions, discharged either directly or through wastewater treatment plants.

Figure 3 shows that another source of contamination in aquatic systems comes from veterinary activities, which generate solid and liquid wastes. Solid wastes are deposited, while liquid wastes, including the liquid separated from solids, are discharged into groundwater or surface water, often via wastewater treatment plants.

### 2.3. Production and Consumption of Pharmaceutical Products

Although extensive data exist on PhP consumption in different countries, specific consumption figures cannot yet be established [56], as many drugs are sold without a medical prescription, making accurate consumption assessment difficult [7]. In Great Britain, for instance, 3000 drugs are registered to be sold without medical recommendation. In 2002, worldwide antibiotic consumption was between 100,000 and 200,000 tons [57]. In the same period, the production of paracetamol in the USA was about 5790 tons [58], while in France, in 2005, it was 3303 tons. In South Korea, a chlortetracycline consumption of 2763 tons has been reported for veterinary applications. A total consumption of 50 drugs used in Great Britain was reported to comprise about 6000 tons in 2002, of which paracetamol consumption alone contributed 3500 [2]. Worldwide antibiotic consumption in 2015 amounted to 8 billion doses, calculated with reference to the minimal maintenance dose, meaning a proportion of 65% of the level consumed in 2000 (21.1 billion doses) [57]. On the European market alone, 3000 PhPs are available [3]. Currently, there are about 4000 pharmaceutical ingredients available on the market, with consumption comprising about 100,000 tons/year [53,59].

### 2.4. Concentrations of Pharmaceutical Contaminants in Natural Water

Research has established that not all PhPs are similarly metabolised. For example, a proportion of only 40% of amoxicillin (antibiotic) is metabolised. These values of other drugs are listed as follows: atenolol (betablocker)—10%; carbamazepine (antiepileptic)—97%; cetirizine (antihistaminic)—50%; diclofenac—85%; erythromycin (antibiotic)—75%; ibuprofen (analgesic)—90% [60].

For this study, 42 PhPs were selected. These have been categorised according to their applications in therapeutics and have been grouped as follows: antibiotics, analgesics and anti-inflammatories, betablockers, antihypertensives, drugs for reducing blood pressure, cardiovascular, antiallergic, anticonvulsive, antidepressive, antiepileptic, antihistaminic, antipsychotic medications, agents for pain reduction, agents for lipid reduction, and NSAIDs.

These PhCs were selected based on the interest that has been shown by various countries in monitoring the most hazardous micropollutants that are present in surface and ground water. The data have been collected from countries in Europe (Germany, France, Great Britain, Spain, Portugal, Italy, and Serbia) and the other continents (South Africa, Australia, and USA).

PhP consumption may be influenced by socio-economic conditions. In Greece, during the 2010–2014 crisis, drug consumption—particularly that of psychoactive drugs—very much increased. Indeed, wastewater were found to contain antipsychotic compounds at concentrations 35 times higher than the admissible dose, and antidepressant compounds at concentrations 13 times higher than the admissible dose [61]. Moreover, drug consumption varies seasonally, with higher usage in winter when respiratory infections increase [62]. PhP consumption is also higher for wealthy populations in comparison with poorer populations [63].

A significant amount of PhPs are consumed in the agricultural sector, applied for both plant growth and animals. Agricultural parasites are controlled by insecticides. Veterinary PhPs are administered orally and are excreted directly into water or through animal feed, either for the prevention or treatment of various infectious and non-infectious diseases. Antibiotics (for instance, tetracycline), anti-inflammatory drugs, and supplementary feed additives are widely used. Recently, antibiotics and hormones used for stimulating development have been reduced in Europe and North America. Animal growth in households could cause serious problems for the environment, since wastewater from animal waste deposits are usually discharged without treatment.

### 2.5. Monitoring and Statistics of Water Contaminants—General Procedures

Although they are not a primary mode of assessing pharmaceutical pollutants, statistical tools play an important role in water control and monitoring and are considered key components in water quality analyses [64]. A very interesting approach that is frequently encountered in the specialized literature is represented by multivariate statistical techniques (MSTs)—utilised in concrete case studies like that reported by Wang et al. [65]—where principal component analysis, discriminant analysis, and factor analysis are used in particular. MSTs are methods used to analyse data that involve two or many variables simultaneously, allowing researchers to examine relationships, patterns, and structures that cannot be understood by simply looking at each variable separately. MSTs are applied when the variables may be correlated or interact with each other; the goal here is to reduce their dimensionality (summarizing many variables into fewer ones) or to model and predict outcomes based on multiple inputs. The main categories of MSTs include exploratory techniques (principal component analysis (PCA), factor analysis, cluster analysis), inferential techniques (multivariate analysis of variance (MANOVA), discriminant analysis), and predictive techniques (multiple regression, canonical correlation analysis, or partial least squares (PLS)). Of these MSTs, cluster analysis and factor analysis have been used by Ismail et al. [66] in order to detect the pollution sources and the main factors which could affect the water quality in the Danube River during various sampling and monitoring periods. Water quality can be determined by its physicochemical and biological properties. The authors of [67] utilise cluster analysis, multivariate statistical methods, principal component analysis, and factor analysis for the interpretation and assessment of water quality data. These statistical techniques are very important when seeking to identify pollution sources and when building a better understanding of changes in the quality of surface water. This involvement of multivariate statistical methods in data monitoring provides valuable information about the chemical composition of water. This is because MSTs are important tools in the analysis and interpretation of complex data matrices, allowing us to better understand water quality and ecological statuses in studied systems; moreover, they enable us to verify temporal and spatial variations caused by natural and anthropogenic factors linked to seasonality and identify the possible factors/sources that influence water systems. The authors of [68] conducted an assessment of spatial variability and determined the main contaminators of surface water quality using multivariate statistical analysis techniques, considering principal component analysis and cluster analysis. When one utilises monitoring techniques that accurately assess water quality and determine the effects of pollution using multivariate statistical methods and water quality in water management, one finds important instruments to evaluate the processes controlling water chemical composition and overall water quality status. This idea is proved by da Silva et al. [69], who utilised a holistic approach for determining water quality conditions and their related effects. MSTs were also used to assess the quality of surface water in [70], where temporal and spatial water quality variability were used to reveal the characteristic properties and pollution indicators of the studied area. Many other works [71,72,73,74,75,76] have presented water evaluation approaches using MSTs; these are followed by case studies that validate the utilised statistical instruments, and these mathematical approaches are successfully applied.

One of the most useful combinations for realizing rigorous monitoring and the assessment of water quality, based on the spatiotemporal analysis of water quality for surface water control, is provided in [77,78,79]. This is also achieved for groundwater in the case studied by Dawood et al. [79], who used MSTs combined with a water quality identification index. MSTs have also been applied to solve similar water pollution problems in [80,81,82]. Nevertheless, statistical tools are not the only focus of water studies; this is particularly the case for studies of surface water. Several other statistical methods and models have made significant contributions to such analyses, as documented in references [83,84,85,86,87,88,89,90,91,92].

In the process of data collection for analyses of water quality, statistical tools have been used and capitalized upon; this is the case in papers [93,94], in book chapters like [95], and in extended works, like [96,97,98].

To obtain an accurate solution regarding the treatment efficiency, an important decisional instrument can be considered ANOVA factorial analysis either with or without interaction. Factorial experiments involving two, three, or several factors realize comprehensive analyses and extend their possibilities to search for proper solutions for many PhCs, using different methods to choose the most adequate way of cleaning wastewater or treating drinkable water; this is highlighted in [99].

### 2.6. Impact of Pharmaceutical Wastes on Environment, Effects on Human Health, and Global Risks

When the chemical content of PhCs contaminating the environment is taken into account [100], one may consider the high quantity of organic substances, like alcohols and ketones, which lead to oxygen depletion and eutrophication (i.e., algal bloom); they also inhibit toxic compounds (i.e., antibiotics), implying a degree of antibiotic resistance, with negative consequences on human health through the direct consumption of drinkable water. This can also lead to haematological and hormonal imbalances in fish and, through the lack of oxygen, the health of the normal flora and fauna of ecosystems is affected. Slowly biodegradable organic compounds and recalcitrant molecules (aromatic compounds, chlorinated hydrocarbons, etc.) produce disruptions in the immune responses of aquatic fauna. Considering the toxic effects on aquatic flora and fauna, pharmaceutical contaminants, including some kinds of antibiotics, have been reported to alter the growth of phytoplankton, while some antibiotics and anti-inflammatory drugs have great negative effects on microorganisms. A pronounced negative impact on marine amphipods and several other aquatic invertebrates is associated with ocean-dumped PhPs residues. Such activities result in many effects of PhPs on the environment and ecosystems; they have eco-toxic consequences for microbial communities and for human health [101].

In [102], it was remarked that the effects caused by drugs in the aquatic area have not been adequately understood or interpreted yet. It has been confirmed that ecotoxicological models do not completely present the effects of pollutants on organized aquatic systems, and ecotoxicological tests are very important forms of analysis in the description of the destructive effects of active contaminants on organisms’ lives. Therefore, specialised studies for the assessment of ecological risks are necessary for highlighting the negative effects related to the occurrence, bioaccumulation, and processing of drugs in the environment, because our knowledge of their pharmacology remains poor.

The authors of [37] report on the health impacts for aquatic organisms caused by PhP contaminants that can bioaccumulate in the aquatic food chain and then transfer from different environmental compartments to humans. In this work—and in several other specialty studies—the need to develop appropriate research for assessing the potential risks of PhP pollutants for biodiversity has been highlighted.

The risks associated with PhC discharge in water are related to aquatic life. In the review presented in [103], a sequence of studies on the effects of this kind of contamination are mentioned in relation to water bodies; their harmful impact on aquatic ecosystems is proven, and this is followed by proposed solutions for mitigate their risks and suggestions for heling aquatic ecosystems to be healthy and thriving. A recent study [104] deepens our understanding of the effects of PhCs on mussels’ digestive glands. It is impressive how much can be achieved by cooperation among practitioners at a high scientific level, with specialized laboratories with many instruments, across many research domains; here, such a collaboration was able to demonstrate the disruptions produced by the accumulation of special types of pharmaceuticals in mussels.

Regarding the impact of PhCs, as it has been analysed in comprehensive reviews, we also mention [105], where the authors presented their findings on the effects of PhCs on aquatic and terrestrial wildlife, accounting for potential risks for human health. In [106], PhP wastes are described as destructive factors, with harmful impacts on entire environments and, consequently, on humans; the authors also list a lot of the diseases that are produced by PhPs. The environmental impacts of PhP pollutants in aquatic systems—taking into account the substances detected in surface and groundwater—are reviewed in [107]; their goal was to build a solid basis for the proposal of some remediation technologies. Hence, it is clear to see that the worldwide effects, impacts, and risks of contamination require the implementation of global measures if we are to diminish and remediate this difficult problem.

A selective list of some PhPs or classes of PhPs, together with their impacts and risk assessment for human health and aqueous systems, is given in Table A2 (Appendix A.2), with corresponding references from the literature.

## 3. Removal of Pharmaceutical Contaminants from Aqueous Effluents

**Figure 4:**
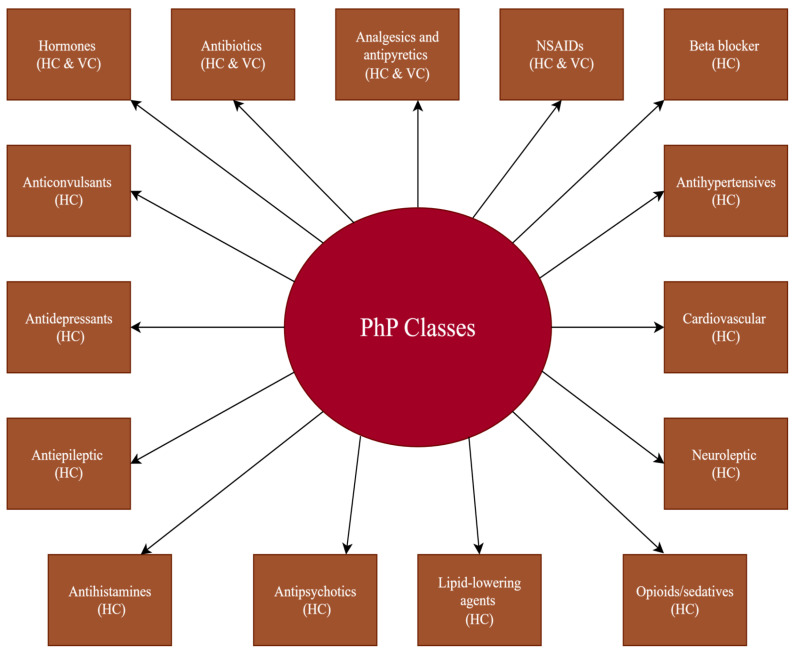
The classes of PhPs found in water needing removal; human consumption (HC); veterinary consumption (VC).

**Figure 5:**
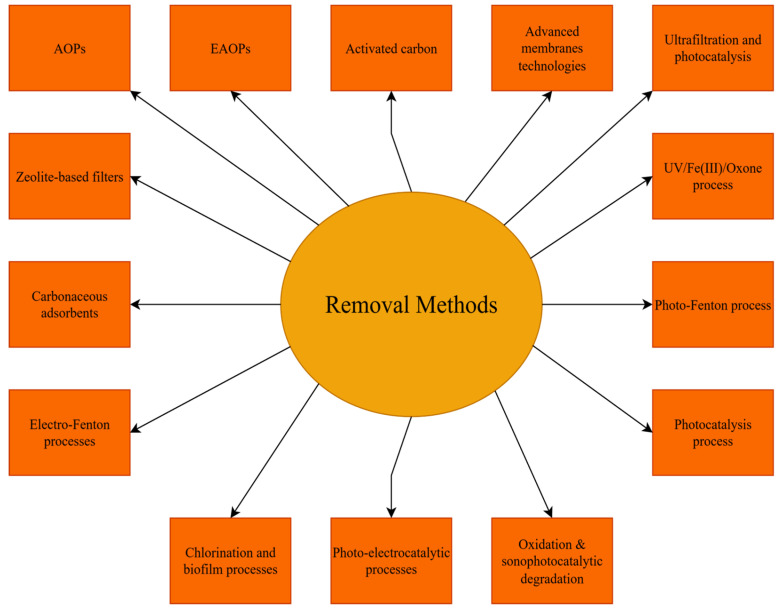
Removal methods for specific PhCs discussed in this section; AOPs—advanced oxidation processes; EAOPs—electrochemical advanced oxidation processes.

To attain a general view on the content of this section, we firstly summarize (in Figure 4) the classes of PhCs, the removal of which is analysed in detail in the works cited in the following text. A list of the most representative PhPs found in water sources, for which disposal methods are reviewed in this section, is given in Table A3 (Appendix A.3). The removal methods discussed in this section are displayed in Figure 5. A selective list of the removal and cleaning efficiencies of the presented methods is displayed in Table 1.

Conventional wastewater treatments are characterized by PhC removal efficiencies ranging from less than 20% to more than 90% [1], values varying function of several factors, while advanced wastewater treatment processes, such as reverse osmosis, ozonation and advanced oxidation technologies, can achieve higher removal rates for pharmaceuticals. Conventional drinking water treatment processes have turned out to be largely ineffective in pollutants removal, with an efficiency of approximately 50% for pharmaceuticals [1]; meanwhile, advanced water treatment processes, such as ozonation, advanced oxidation, activated carbon, and membranes (e.g., nanofiltration, reverse osmosis), are able to achieve higher removal efficiencies (above 99%) for targeted PhPs. This has been shown in various studies in the published literature. In this section, several innovative cleaning or treatment methods are presented and discussed.

### 3.1. Adequate Methods to Remove Pharmaceutical Pollutants

#### 3.1.1. Advanced Oxidation Processes

An overview of the use of advanced oxidation processes for the degradation of nonsteroidal anti-inflammatory drugs is provided by Silva et al. [108], who underline the hazardous effects of these compounds in conventional treatment methods. Although the concentration of these drugs is not regulated by the legislation that is in force to regulate water quality, their presence is of significant concern, as they are persistent and biologically active contaminants. Despite the efficiency demonstrated by advanced oxidation processes (AOPs), hybrid methods based on an integrated reactors containing AOPs combined with membrane filtration provide increasing efficiency in NSAID degradation. However, such hybrid approaches involving simultaneous membrane filtration and AOPs are quite dependent on membrane characteristics. As a result, the evaluation of new materials for membrane construction with adequate characteristics should be taken into consideration, as recommended in [109].

In the same context, a critical review on the advanced oxidation processes applied in wastewater treatment for the degradation and destruction of persistent organic pollutants during 1990–2012 is provided by Oturan and Aaron [110]. Because of the numerous pollutants that are generated by anthropic activities, it is urgent to propose and apply highly efficient techniques for the protection of natural water.

The main principles, advantages, and disadvantages of such approaches are critically analysed for various types of AOPs, including peroxonation, photo-Fenton (H_2_O_2_/Fe^2+^/UV) reaction, heterogeneous photocatalysis (TiO_2_/UV), and electrochemical AOPs. Among these techniques, photochemical AOPs are generally simpler, cleaner, relative more cost-effective, and more efficient than classical chemical AOPs. The use of AOPs is considered to be the best method for ensuring rapid photodecomposition and for optimising the mineralization yield of various contaminants.

A more efficient alternative to the classical photo-Fenton process is the solar photo-Fenton AOP, which is cost-effective and has low energy consumption levels. It can be used for the removal of various pollutants that are present in natural or wastewater until they reach complete mineralization. Moreover, heterogeneous photocatalysis is mainly based on titanium oxide semiconductors, made of a chemically highly stable material, which is very easy to produce, biologically inert, and endowed with energy characteristics that are comparable to those of solar photons.

The combination of Fenton-type reaction with ultrasounds resulted recently developed rapid sonochemical methods, which have promising prospects for decontamination applications.

Ozonation involves high energy consumption and exploitation costs, and it cannot be applied at a large scale, while the secondary products from the process cause serious environmental problems. Advanced oxidations (H_2_O_2_, UV, O_3_, etc.) although are highly efficient in PhC removal, form byproducts that are sometimes more toxic than the removed pollutants, and they require complicated plants and have high utilization costs.

**Figure 6:**
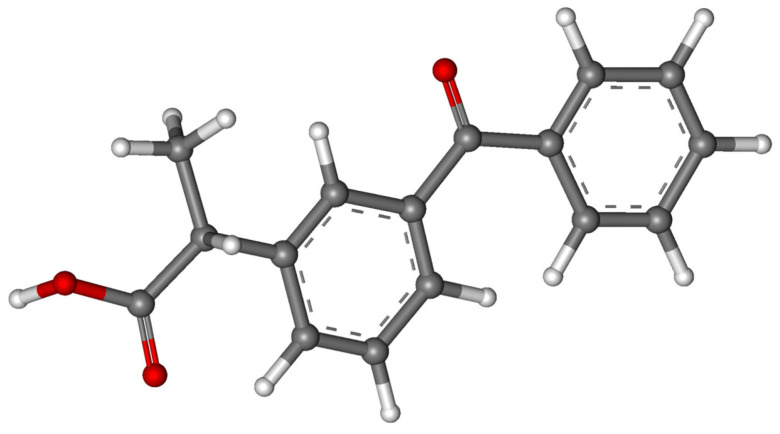
Three-dimensional theoretical structure of ketoprofen [112]; grey spheres for carbon; white spheres for hydrogen; red spheres for oxygen.

Mei et al. [111] reported on the thermodynamics and reaction mechanisms of the ozonolysis of ketoprofen (Figure 6) in polluted water. The reaction of ozone with water under ultraviolet irradiation can produce hydroxyl radicals, which can further enhance the degradation process. The ozonation of ketoprofen is not influenced by the solution’s pH.

The toxicity of the oxidation substances produced by degradation reactions indicates that many of the degradation compounds are not dangerous, but a few aromatic products are still toxic and have to be eliminated.

**Figure 7:**
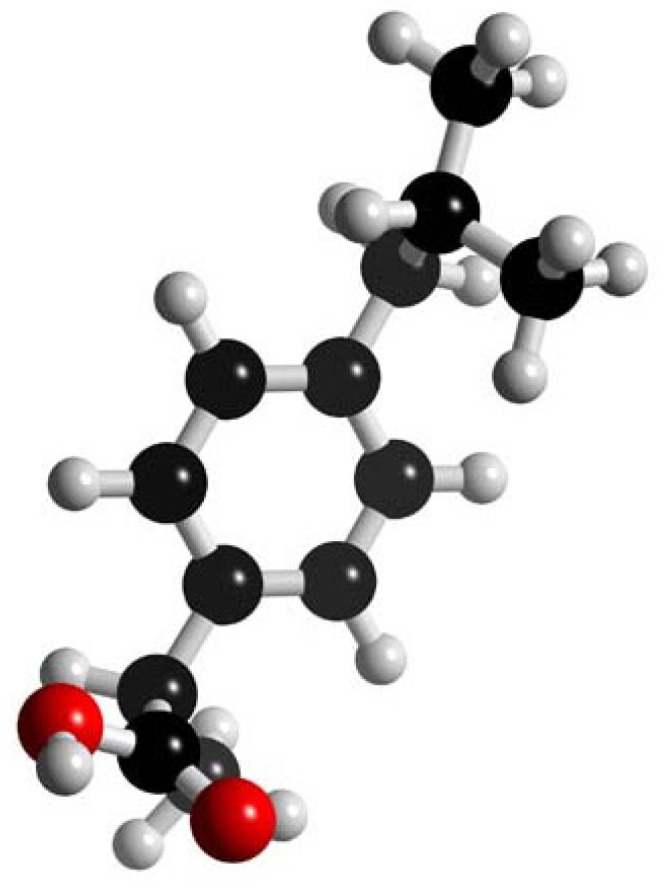
Theoretical structure of ibuprofen [115]; black—carbon; white—hydrogen; red—oxygen.

Rao et al. [113] applied a synergistic UV/Fe(III)/oxone process for the degradation of ibuprofen (Figure 7); this process was compared with the sole-UV, UV/Fe(III), Fe(III)/oxone, and UV/oxone processes. Nineteen intermediate products were identified; meanwhile, it was found that decarboxylation and hydroxylation are the major reactions involved in ibuprofen destruction. Although a toxic compound was identified (4-isobuthylacetophenone), its complete destruction was expected, since its concentration decreased after 20 min of the ibuprofen degradation reaction. Solid-phase microextraction coupled with GC/MS has been used for the identification of intermediates and byproducts. The presence of certain inorganic anions like Cl^−^, SO_4_^2−^, and H_2_PO_4_^−^ exerted a significant effect on the photo-oxidative process.

Ibuprofen has been also removed from water by photo-Fenton process; this has been reported on by Loaiza-Ambuludi et al. [114]. This method was used for coupling UVC irradiation and Fenton’s reagent in order to catalyse the in situ generation of hydroxyl radicals, which are able to destroy organic pollutants until total mineralization is achieved. The results of such studies demonstrate that the photo-Fenton process is more efficient than other systems, assuming TOC removal of up to about 90%. Higher mineralization was obtained in the photo-Fenton process as a result of the action of hydroxyl radicals, generated by the Fenton reaction and the enhancement provided by the UVC irradiation. For the destruction of ibuprofen residues in aqueous effluents, it is advisable to conduct coupling with biological or photo-Fenton treatments, reducing the costs that will be incurred when an artificial UVC source is used.

**Figure 8:**
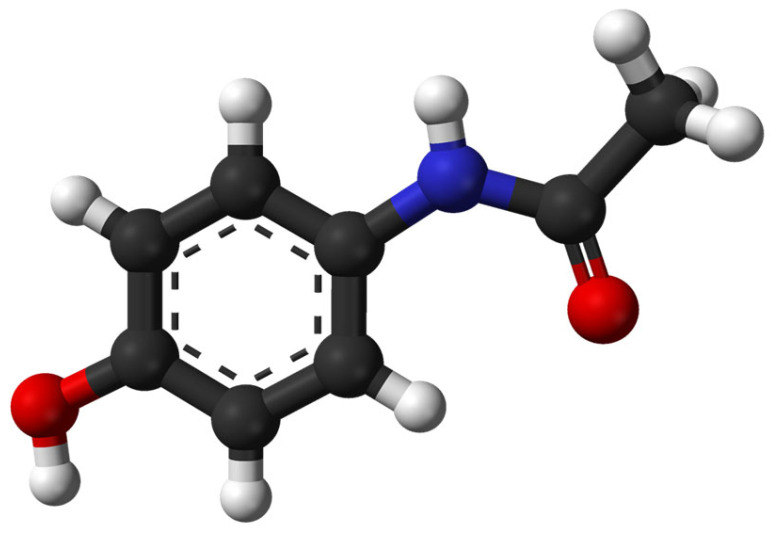
Three-dimensional theoretical structure of acetaminophen [117]; blue—nitrogen; the others—as above.

Luna et al. [116] proposed a method for acetaminophen (Figure 8) degradation by electro-Fenton and photo-electro-Fenton processes using an electrochemical cell with double cathode. The Box–Behnken procedure was used to assess the effects of initial concentrations of Fenton reagents and those of the current density applied. The electro-Fenton method was found to be more advantageous from an economical point of view and can be extended at a large scale.

**Figure 9:**
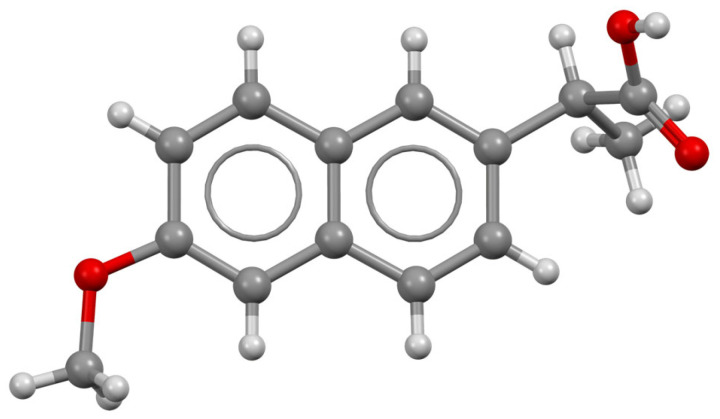
Three-dimensional theoretical structure of naproxen [119]; grey—carbon; white—hydrogen; red—oxygen.

An alternative method for naproxen (Figure 9) removal was conceived by anodic oxidation using a fluorine-doped tin oxide glass (FTO) [118]. By using FTO glass, two anodes were successfully synthetized by doping the glass with platinum nanoparticles: Pt-FTO and Pt/MWCNTs-FTO. The latter anode had multi-walled carbon nanotubes as the support material. Platinum nanoparticles and multi-walled carbon nanotubes have two major advantages, while acting to ensure the effective demineralization of naproxen in aqueous mixtures: lower activation energy in degradation of naproxen; higher adsorption capacity for hydroxyl radicals. As a result, these supporting materials can protect the surfaces of platinum nanoparticles from sintering; thus, they can provide a high number of active sites to interact with naproxen in solution. The degradation efficiency of naproxen is favoured by acidic pH values. The presence of humic acid inhibited the degradation of naproxen in this multi-component system. The efficiency of this method is compared with that of other techniques, such as biological treatment, sorption on activated carbon or metal–organic frameworks, photolysis, and the combination of ultrasound irradiation with photo-Fenton reagents. Based on this comparison, two improved degradation methods have been proposed for naproxen.

The removal of ibuprofen from water using a homogeneous photocatalysis process has been reported by Loaiza-Ambuludi et al. [114], and the degradation of naproxen using such a technique was reported on by Coria et al. [120]. The nature of the anode material used has an important role in the degradation mechanism, and different electrochemically advanced oxidation processes have been comparatively analysed: electro-oxidation with electro-generated H_2_O_2_; electro-Fenton and UVA photo-electro-Fenton.

Degradation of ibuprofen and naproxen by a three-dimensional electro-Fenton system has been studied by Mohammadi et al. [121]. Response surface methodology was applied to assess the interaction effects of some operating variables, like pH, current density, reaction time, and pharmaceutical concentrations.

**Figure 10:**
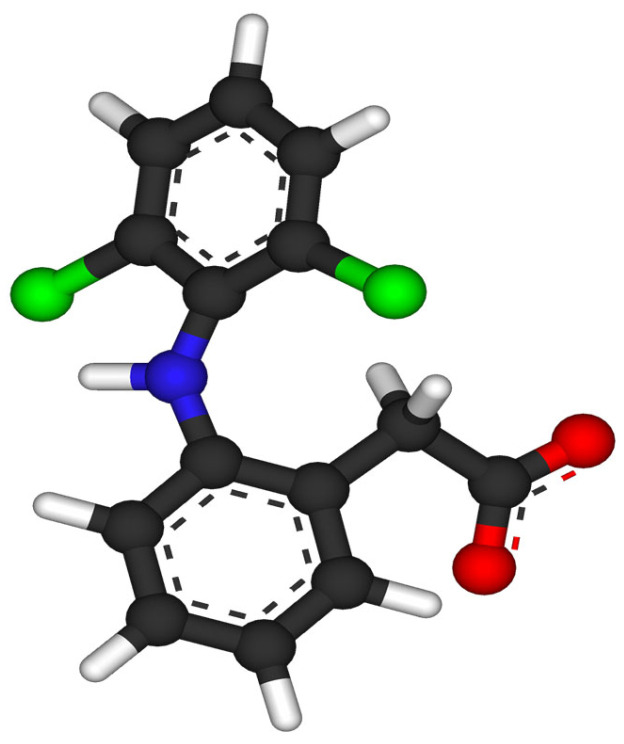
Three-dimensional theoretical structure of diclofenac [123]; black—carbon; white—hydrogen; red—oxygen; green—chlorine; blue—nitrogen.

Diclofenac (Figure 10) has been removed from wastewater through electrocatalytic and photo-electrocatalytic processes by using mesostructured lead dioxide grown on titania nanotubes (NTs) [122]. When such mesostructured materials were exposed to visible light irradiation (365 nm) in the photo-electrocatalytic experiments, only the TiO_2_ NT_s_:PbO_2_ anode showed significant enough photoactivity for the degradation of diclofenac from the solutions. At the same time, this pharmaceutical oxidation seems to be much more efficient for decreasing chemical oxygen demand (COD).

Diclofenac and ibuprofen have been removed from water using scrap printed circuit boards [124]. According to waste-to-resource technologies, electronic waste can be transformed into efficient instruments for environment remediation. In this context, such electronic waste has been reused to prepare heterogeneous H_2_O_2_ activators, such as non-carbonized catalysts obtained under air combustion and a carbonized catalyst prepared under nitrogen pyrolysis. These two contaminants were used as model pharmaceutical pollutants to assess the efficacy of the newly synthetized catalysts in the Fenton oxidation process. The effects of the experimental factors (such as catalyst/H_2_O_2_ dosage, initial concentrations, and solution pH) on the removal rate were optimized. It was established that copper oxide catalysts play a decisive role in the degradation process, and these catalysts have good reusability for five cycles, with low metal leaching. The degradation kinetics followed a pseudo-first-order model.

The enhancement of diclofenac removal from wastewater using a combination of a photocatalyst process and oxidation with hydrogen peroxide was suggested by Tra et al. [125].

Advanced oxidation combined with sonophotocatalytic degradation of ibuprofen was reported by Madhavan et al. [126], where the final products obtained by oxidation of propanoic acid and the isobutyl substituents of ibuprofen have been detected. Both TiO_2_ and Fe^3+^ sonophotocatalyses showed a significant synergy in the degradation of ibuprofen when compared with individual sonolysis and photocatalysis.

A review on the use of advanced oxidation to remove pharmaceuticals and personal care products has been elaborated by Krishnan et al. [127], who highlighted the limited and widespread metabolic intake capacity of these materials. To ensure their effective elimination, a series of AOP methods were applied: ionizing radiation, electrochemical oxidation, sonication, etc. Different classes of PhPs, like analgesics, disinfectants, and antibiotics have been completely removed. In ultrasonication, an increasing ultrasound power density favoured PhCs elimination; meanwhile, in electrochemical oxidation, a higher current density and the nature of the anode were the most significant factors influencing the degradation process. A promising direction for this research is to use hybrid methods, combining biological methods with ionizing irradiation in the development of more efficient catalysts.

The detection and removal of personal care products (PPCPs) and pharmaceutical contaminants by electrochemical advanced oxidation processes (EAOPs) have been reported on by Lozano et al. [40]. It was found that the main methods used for PPCPs removal are electro- and photo-electro-Fenton, solar photo-electro-Fenton, and sonoelectrochemical processes. Among the most representative pollutants, three classes have been selected and tested: antibiotic, anti-inflammatory, and antifungal compounds. The most versatile processes applied for removal of these emerging contaminants are the photo-electrocatalysis and electro-Fenton processes.

Feng et al. [128] reviewed on NSAIDs removal using electrochemical AOPs. Indeed, the low removal efficiencies of these contaminants by conventional wastewater cleaning plants require a more efficient technology. Currently, advanced oxidation processes have become a hot research topic; these technologies have been shown to efficiently oxidize most organic pollutants to reach mineralization to inorganic carbon (CO_2_). Both anodic oxidation and electro-Fenton processes have demonstrated good potential at the lab-scale level for the abatement of pollution caused by residual pharmaceuticals in wastewater.

The efficiency of several methods presented in this subsection are displayed in Table 1. A great value of removal efficiency is not the single indicator of solution efficacy, since in many cases the byproducts represent another difficult problem (almost general for AOPs).

**Table 1:** Cleaning or treatment efficiency of the methods discussed in Section 3.1.1.

PhC/Class of PhPs	Cleaning/Treatment Method	Efficiency	Citation
NSAIDs	Membrane filtration and AOPs	90–94%	[108]
NSAIDs	EAOPs	>90%	[110]
Ibuprofen	UV/Fe(III)/Oxone	~100%	[113]
Ibuprofen	photo-Fenton	96%	[114]
Ibuprofen	Fenton-based AOPs	66%	[124]
Ibuprofen	Sonophotocatalysis/TiO_2_ or Fe^3+^	~100%	[126]
Acetaminophen	electro-Fenton & photo-electro-Fenton	100%	[116]
Naproxen	electrochemical oxidation	~100%	[118]
Naproxen	EAOPs	91%	[125]
Ibuprofen	3D electro-Fenton	93.51%	[121]
Naproxen	3D electro-Fenton	98.14%	[121]
Diclofenac	electrocatalysis & photo-electrocatalysis	>90%	[122]
Diclofenac	Fenton-based AOPs	86%	[124]
Diclofenac	AOPs (UV/TiO_2_/H_2_O_2_)	100%	[125]

#### 3.1.2. Cleaning Technologies for Pharmaceutical Contaminants Removal from Aqueous Effluents

Technologies involving mechanobiological purification approaches that are utilised at existing treatment plants, even those with a tertiary level of use, were not designed to eliminate PhCs from waste and drinkable water. Tertiary purification is different from advanced purification. The first level involves processes of denitrification and dephosphorization, fulfilling the conditions stipulated by European legislation; meanwhile, advanced treatment/purification processes include reverse osmosis, ozonation, or other advanced oxidation technologies.

Among these techniques, adsorption on solid particles in suspension and biodegradation contribute to efficient removal of contaminants. Biodegradation is produced mainly when PhCs are dissolved, while the adsorption is involved if the particles are in suspension. Electrostatic interactions of a with hydrophobic character occur between these materials and PhCs.

The hydraulic and solid retention time of antibiotics plays the most important role in treatments with activated sludge [129]. PhC (mainly tetracycline) decomposition is assisted by an increase in retention time. PhC molecules with long and multi-branched chains are less biodegradable [130]. Saturated aliphatic and aromatic compounds with sulphur or chlorine in the substituent position are also resistant to biodegradation [131].

The age of the sludge is 2–3 days for the degradation of sulfamethoxazole, aspirin (acetylsalicylic acid), and ibuprofen, and 5–15 days for diclofenac and roxithromycin. Diazepam and carbamazepine remain unchanged, even after more than 20 days [132].

Advanced treatments have better outcomes than conventional technologies, and the efficiency of PhC removal can reach 100%.

The principal technological operations of advanced cleaning/treatments are advanced oxidation (photolysis, ozonation, UV, and H_2_O_2_), micro-, ultra-, and nanofiltration, reverse osmosis, and adsorption on activated carbon.

Some water-soluble organic compounds with polar characteristics (for instance, sulfamethoxazole) are not easily adsorbed on activated carbon. Some organic compounds, such as PhCs in water, can be efficiently removed by advanced oxidation techniques. The removal efficiencies of PhCs by these methods are inferior to those achieved using ozonation [133]. The microfiltration and ultrafiltration membranes used in such techniques do not have the pore dimensions required to retain the big molecules of such pollutants [134].

Water with high salt concentrations and PhCs with small molecular masses can cause big problems for membranes at high pressures. Artificial infiltration in soils or in the beds of rivers can be a pretreatment solution for drinkable water. The artificial infiltration of the water leads to a self-purification phenomenon in soil. Such an operation can be associated with the slow filtration applied to drinkable water. This practice is not adequate for antiepileptic PhPs [135] or for polychlorinated bisphenols.

Nanofiltration and reverse osmosis have been shown to remove over 85% of PhCs from underground water [136]. Adsorption on activated carbon (powder or granulated) is frequently applied to remove organic micropollutants from wastewater and especially from drinkable water. Photocatalysis, although it has efficiency in PhC degradation applied on a large scale, exhibits high energy consumption, has huge costs, and the production of secondary products limits its use.

An advanced membrane technology proposed by Qurie et al. [137] has been successfully applied for the removal of naproxen, diclofenac, and a heavy metal—a chromium (VI) composite material. An efficient adsorbent has been prepared using a cationic surfactant based on octadecyltrimethylammonium salt and montmorillonite—a negatively charged clay mineral. The efficiencies of three technologies have been determined and compared for the removal of diclofenac and naproxen: ultrafiltration membranes, activated carbon, and reverse osmosis. While the first method gives results that are not satisfactory, the last two present good efficiencies or near 100%, with pore sizes less than 0.5 nm. The results demonstrated that the micelle–clay complex is more efficient in retaining the adsorption of both organic and inorganic pollutants in comparison with activated carbon.

Innovative technological solutions for the removal of pharmaceutical contaminants from water sources have been proposed by Meghea et al. in [99]. Their study discusses the rehabilitation of existing treatment plants and wastewater cleaning plants through updating their extant technologies using advanced treatment operations: adsorption on activated carbon, nanofiltration, and reverse osmosis. Other studies have reported on the typical technological schemes that are used in advanced surface water and underground water treatment approaches, processing drinking water supplies or water supplies with very high water hardness. The authors of [138] describe a design procedure for improving some existent plants, using a special kind of membrane, which is considered a viable route for their rehabilitation; the approach involved low construction and operating costs. The study also presents calculations of the development process and numerical examples.

### 3.2. Studies Focused on One or Two Specific Pharmaceutical Contaminants

The removal of ketoprofen from synthetic surface water with humic acid content has been analysed by Szymanski and Mozia [139] by coupling ultrafiltration and photocatalysis within a new photocatalytic membrane reactor. The treated water was supplied to a feed tank equipped with an ultrafiltration membrane; then, it was transferred to a UVA-irradiated labyrinth flow photoreactor. A photocatalyst, titanium dioxide, was added to the feed solution. The positive influence of aeration on ketoprofen decomposition was observed. An advantage of the reactor configuration is the separation of the photoreactor unit from the membrane tank, with a role of protecting the membrane from the destructive action of oxygen radical species and of UV radiation. As a result, this type of photoreactor allows the irradiation area of the photocatalyst to be extended, along with the time spent in contact with pharmaceutical pollutants. These factors contribute to the increase in the treatment’s efficiency. However, further optimization of the process is needed in order to facilitate the transfer of data from the laboratory to large-scale applications.

Smiljanić et al. [140] showed the influence of inorganic anions on the adsorption of ibuprofen and naproxen through the use of zeolite-rich composites that had been modified in two ways: monolayer and bilayer surfactant coverage. The effect of the working conditions on the adsorption of these drugs was controlled in a buffer solution. The Langmuir model demonstrated a higher adsorption capacity for the composite involving the bilayer surfactant at the clinoptilolite surface, indicating that—aside from the hydrophobic partitioning that was specific to the composites with a monolayer—ion exchange was a verified adsorption mechanism. In this way, such functionalized zeolites could serve as low-cost adsorbents for water treatment processes. If one compares the adsorption behaviours of these natural adsorbents, it is obvious that the hydrophobicity of the drugs strongly influences their adsorption capacity.

Cuomo et al. [141] proposed the combination of a zeolite-based filter with the reduced content of powdered activated carbon for the removal of eleven micropollutants from wastewater, contributing to saving costs and a reduction in CO_2_ emissions.

A complete assessment of the environmental impact and application potential of water hyacinth for the removal of organic pollutants is provided by Ishak et al. [142]. The authors showed that both the naturally occurring and the chemically engineered forms of water hyacinth [142] can cause difficulties in water treatments. The primary conditions for water hyacinth to be used as an adsorbent are of high importance: there is no demand for any specific operation, equipment, or reagents to be used in the cleaning process. It has been shown that water hyacinth can absorb organic compounds from wastewater, which is an unusual feature of the invasive plant in water. Since the water hyacinth plays an important role for aquatic life survival, adequate research is essential to find an equilibrium between water remediation and the health of marine species. The addition of water hyacinth waste in the manufacturing of eco-friendly biochar is advantageous as an alternative for emerging strategies in solid waste management, and it may help in the removal of organic pollutants.

An ecotoxicological assessment has been provided by Priyan and Narayanasamy [143] for the removal of ibuprofen and sulfamethoxazole from water, using corn starch nanoparticles as bio-adsorbents. The removal of ibuprofen followed a Temkin isotherm model, while for sulfamethoxazole, a Langmuir isotherm model was verified. Toxicological tests were performed on zebrafish organisms, and a lethal dose was provided.

NSAIDs, such as naproxen and ketoprofen, may be removed from river water using low-cost carbonaceous materials as adsorbents [144]. The adsorption process is favoured by low values of temperature and pH, and by high ionic strength. The porous adsorbents are more effective in the removal of pollutants. Moreover, the use of natural river water may contribute to the efficient removal of naproxen and, especially, ketoprofen. One may observe that the aqueous matrix is a major factor in determining the adsorption process. In cases of drug contaminants, the use of natural river water leads to a larger adsorption capacity, due to the presence of solid matter in suspension that plays the role of a co-sorbent for the removal of pharmaceutical pollutants. However, the interpretation given by the authors—where they assert that the influence of temperature on adsorption capacity is a result of the increasing drug solubility and stronger interactions between the drug molecules and the active sites of the adsorbent surface—is rather wrong. The actual cause of the decrease in the adsorption capacity is adsorption being an exothermal process. This is the real reason why adsorption is not favoured by increasing temperature.

An analytical review was provided by Rozaini et al. [145] on advanced adsorptions of NSAIDs for improving offline and online preconcentration techniques; the last online technique utilizes less solvent, thus aligning with the Green Analytical Chemistry Initiative.

The uncontrolled use of NSAIDs as analgesics for various conditions has perpetuated their presence in the environment, with concentrations detected in natural water being around 10^−1^ mg L^−1^, potentially threatening human health. As consequence, continuous control in pollution monitoring is compulsory for preventing any hazardous effects. In this context, a series of preconcentration techniques have been proposed, reflecting the increasing demand for a more performant method that can obtain optimal results (relative recovery > 70% and detection limit of 0.1 ng/mL). Other preconcentration methods are discussed. It was found that the multivariate approach is better than the conventional method in developing offline preconcentration techniques.

A novel three-dimensional electro-Fenton system was developed by Mohammadi et al. [121] for the degradation of anti-inflammatory pharmaceuticals, being focused on modelling and degradation pathways under the action of iron-coated nickel particles as catalytic electrodes. In a similar context, a novel stretchable carbon nanotube/Ni@TiO_2_: W photocatalytic composite was used for the removal of diclofenac from drinking water [146].

Naproxen has been shown to be destroyed by chlorination and biofilm processes, as conducted by Boyd et al. [147]. A bioreactor was used to simulate an aerobic biofilm that is specific to wastewater treatment systems. It was observed that naproxen was not biologically degraded; however, when in contact with free chlorine, naproxen products can adversely affect the biofilm process. The formation of reaction products can vary depending on the characteristics of the water or wastewater and the treatment operating conditions.

The mass transfer and thermodynamic parameters of the adsorption of naproxen sodium onto activated carbons obtained from white and black polymeric waste have been evaluated at various temperatures by Sarici-Özdemir and Önal [148]. Specific parameters like the intraparticle diffusion rate constant and the film and pore diffusion coefficients have been followed as functions of temperature, while the adsorption data have been checked using Langmuir isotherm and adequate kinetic mechanisms.

The disinfection of water contaminated by diclofenac was performed by photocatalysis over In_2_O_3_/TiO_2_ heterojunctions in the presence of *Enterococcus faecalis* bacteria as an activator [149]. These photocatalysts, with different indium amounts, are biphasic samples with anatase TiO_2_ and In_2_O_3_ phases; they show significant light absorption in the visible domain and a large interfacial charge transfer from the conduction band of In_2_O_3_ to the valence band of TiO_2_. Simultaneous decontamination and disinfection processes reveal that 5% heterojunction composites are stronger than the titania activity for diclofenac removal in the presence of bacteria.

Removal of rhodamine B and diclofenac from water has been achieved through the use of iron-impregnated biochar obtained from the waste of black seed pomace under the heterogeneous catalytic activation of persulfate [150]. This novel catalyst was prepared using the simple one-step pyrolysis method, using waste black seed pomace as a carbon precursor. This approach showed superior peroxydisulfate activation for the removal of refractory organic pollutants, including pharmaceutical contaminants such as diclofenac.

Diclofenac can be also removed from water by a smart device based on a positively charged covalent organic composite, magnetically modified by chitosan-coated triazine as a bio-adsorbent [151]. The adsorption data were well fitted on a pseudo-second-order kinetic model and on the Langmuir isotherm thermodynamic model.

Joodaki and Mollahosseini [152] evaluated the efficiency of luffa modified with silver nanoparticles for the removal of ibuprofen from aqueous solutions. The modification of luffa resulted in changing the surface charge, while the number of active sites on adsorbent increased significantly. Luffa sponges have OH groups attached to their surfaces, which become negatively charged in aqueous media, thus creating electrostatic repulsion with anionic ibuprofen; here, adsorption does not work well. However, with the addition of silver nanoparticles to the adsorbent surface, this becomes positive, generating electrostatic attraction with anionic ibuprofen. The adsorption of ibuprofen is due to π → π* and π → metal interactions between silver cations and the aromatic ring of ibuprofen.

Ahmad [153] recently drew attention to the fact that many countries face water scarcity as a result of increasing pharmaceutical micropollutant concentrations in aqueous effluents; they proposed the reuse of agro-waste-based adsorbents. These are low-cost, renewable, and sustainable solutions for eliminating ibuprofen and carbamazepine from wastewater. Agricultural wastes are solid residues that remain from agricultural activities. Lignocellulosic residues contain hemicellulose, cellulose, lignin, pectin, starch, and sugars. Their complex structure makes them difficult to degrade, but they can be used as efficient bio-adsorbents of both organic and inorganic pollutants. A good adsorbent should have various physicochemical and technical features: high adsorption capacity, cost-efficiency, ability to be regenerated, mechanical integrity, and chemical stability. However, the sustainability of these agro-waste adsorbents cannot be optimized without many regeneration studies.

Amaral et al. [154] reviewed the removal of organic contaminants from water effluents in mixotrophic mode by phycoremediation technology using the microalgae of the *Chlorella* genus. The study involved the bioaccumulation, bio-adsorption, and biodegradation processes. Applications can be extended to the removal of organic contaminants from body care products, new medications, veterinary drugs, and cleaning products.

A promising approach for the removal of NSAIDs from water was proposed in [155], using new hydrophobic deep eutectic solvents, namely decanoic acid and 2-pentanol, which were functionalized onto magnetite nanoparticles in order to obtain an efficient adsorbent for diclofenac and ibuprofen. The optimized adsorption conditions were established using the Taguchi experimental model and characterization was achieved through adequate advanced analytical techniques. The adsorption process was attributed to hydrogen bonding and hydrophobic interactions, resulting in multilayered adsorption. This material has shown to be a promising nanocomposite for naproxen and diclofenac removal from water, being stable and having low toxicity according to the green chemistry principle.

### 3.3. Research Devoted to the Removal of Pharmaceutical Contaminant Classes

Noutsopoulos et al. [156] highlighted the role played by activated carbon and disinfection process on the removal of NSAIDs and endocrine-disrupting chemicals discharged in secondary effluents.

A recent study reported on the removal of pharmaceutical contaminants present in urban wastewater, produced by a bio-electrochemical process conducted at a pilot scale [157]. The coupled bio-electrochemical treatment system was obtained by a combination of an electro-oxidation process with a microalgae-based system. This procedure showed removal efficiencies above 80% for anti-inflammatory, analgesic, and hypolipidemic drugs; meanwhile, for antiepileptic drugs, the removal efficiency was between 40% and 70%.

Liu et al. [158] reported on the simultaneous removal of diclofenac, ibuprofen, and ketoprofen by electrocoagulation–flotation with a cationic surfactant, cethyltrimethyl ammonium bromide (CTAB), as a collector. The removal of multiple NSAIDs in hospital wastewater was found to be significantly lower, as a result of interference with oils and other hydrophobic contaminants.

A series of 14 perfluoroalkyl compounds have been used for the removal of 46 PhCs in full-scale water treatment plants [159]: 20 antibiotics, 7 anthelmintics, 3 beta-blockers, 8 nonsteroidal anti-inflammatory drugs, and others. It was found that removal efficiency in field-scale drinking water treatment plants with active carbon process increased while the perfluorocarbon chain length increased. However, only six pharmaceuticals were detected in drinking water samples: metformin, caffeine, crotamiton, carbamazepine, sulphametazole, and fenbendazol. The incomplete removal of some pharmaceuticals, such as crotamiton and metformin, can be explained by changing their molecular structures during oxidative processes.

The removal of a mixture of five anti-inflammatory compounds from the aqueous matrix was performed using a synthetized cross-linked, grafted zwitterionic chitosan derivative [160]. As model pollutants, the following pharmaceutical compounds were selected: diclofenac, ibuprofen, ketoprofen, paracetamol, and salicylic acid. A series of parameters—the initial concentration of the pollutant, the solution’s pH, and the contact time between the adsorptive biomaterial and the tested pharmaceuticals—were found to affect the process. The optimum pH was in the acidic domain (pH = 4), and adsorption was assessed according to the ionic interactions that occurred between the specific functional groups of the drugs and the active sites of the biomaterials. However, these authors also sustain that adsorption is favoured by increasing temperatures, what is not true, since desorption is the main process that takes place at high temperatures.

New biocomposites based on chitosan/polyvinyl alcohol/graphene oxide compounds have been used in the removal of pharmaceutical pollutants from aqueous systems [161]. The following pharmaceuticals were selected As the targeted contaminants: four NSAIDs (paracetamol, diclofenac, ibuprofen, ketoprofen), two antihypertensive drugs (valsartan and irbesartan), and one antiepileptic (carbamazepine). Of these contaminants, the highest adsorption capacity was achieved for diclofenac and valsartan. Though this research group has consistent publications in specialized journals, their affirmation that adsorption capacity rises with temperature is incorrect; adsorption is an exothermal process. However, adsorption is usually only one step in a complex reaction mechanism, of which the slowest reaction is the rate-determinant step. That is why the above-mentioned authors found a maximum adsorption capacity of pharmaceutical compounds on chitosan biocomposites at 55 °C—this was probably a result of an endothermal chemosorption process. At higher temperatures, the desorption process becomes dominant.

In the same topic, it is worth mentioning the paper published by Zaka et al. [162], which focused on the removal of diclofenac and aspirin as persistent pharmaceuticals in wastewater, using a reduced graphene oxide magnetite. Their regeneration study showed that the removal efficiency was still important after three cycles.

Recent findings in micropollutant removal from water using innovative adsorbent materials have been reported by Selvakumar et al. [163] by converting biochar into 3D porous aerogel obtained by a one-step direct carbonization-activation method. There are various micro- and nano-3D materials with potential for applications in micropollutant removal and degradation: 3D aerogels, 3D foams, 2D/3D incorporated nanomaterials, additive 3D materials, 3D materials used for 3D numerical modelling, etc.

Copolymers based on acrylic acid have been used as efficient adsorbent materials for the removal of some pharmaceuticals from medical wastewater [164]. Experiments have been performed for the removal of ibuprofen and diclofenac from aqueous solutions. Acidic conditions seem to be optimal for the removal of these contaminants. Unfortunately, these authors have also made mistaken interpretations referring to the influence of temperature on adsorption capacity, neglecting the exothermal nature of the adsorption process.

Zhang et al. [165] reviewed applications of water-stable metal–organic materials in the removal of pollutants from wastewater. Such materials are applied in separation, catalysis, adsorption, and other water environment remediation areas; it is necessary to protect material structures during their use. The removal properties of drugs, organic dyes, heavy metal ions, and radionuclide contaminants by different organic–metal materials are compared and applied in material design and environment cleaning. For instance, naproxen and ketoprofen have been successfully removed by adsorption using graphene oxide/metal–organic composites, as shown by Sarker et al. [166].

Lin et al. [167] reported on recent updates on the occurrence, fate, and removal technologies of NSAID contaminants. Various cleaning technologies, including biodegradation, adsorption, and advanced oxidation are analysed, highlighting their benefits and drawbacks. In view of future research, they recommend that the following areas are prioritized: the development and application of strategies for producing novel catalysts with enhanced catalytic activity and better stability; new electrodes with superior abilities for the generation of hydroxyl radical species within eco-friendly and cost-effective processes.

NSAIDs have been also removed from water flows by adsorption on ionic covalent organic composites, provided by tricarbaldehyde and diaminoguanidine derivatives [44].

Mlunguza et al. [168] discuss strategies for the removal of NSAIDs from contaminated water sources, including activated carbon, molecularly imprinted polymers, graphene-based adsorbents, sonochemical processes, photocatalytic degradation, and electrochemical methods. Other ecological approaches such as remediated wetlands have shown to exhibit high applicative potential for NSAIDs removal.

Activated carbon has been also used in the disinfection and removal of ibuprofen and ketoprofen from biologically treated wastewater during chlorination, when these contaminants have been found in the effluents even after UV irradiation. However, they have been removed in this system in the presence of H_2_O_2_. NSAIDs compounds have also been removed by adsorption on green magnetic nanoparticles [155].

Other magnetic nanocomposites have been obtained from peanut shells, rice husks, tea waste, curcumin nanoparticles, palm waste, and sunflower head waste [169]. Such wastes facilitate precipitation and co-precipitation processes, calcination, and pyrolysis. They can also be used to increase the efficiency of removing dyes, organic, and pharmaceutical contaminants like rhodamine, methyl blue, promazine, amoxicillin, and ciprofloxacin. The adsorption capacities of magnetic nanocomposites depend on the water treatment process being utilised, as magnetic carbon nanocomposites can enhance the removal of various pollutants from contaminated water. In this respect, dye components are readily adsorbed by carbon nanocomposites derived from agro-waste, such as peeled, fibrous, shelled, and leafy types, as in the case of orange peel powder, walnut shell, etc. A series of magnetic carbon nanocomposites can be used as good adsorbents for water treatments due to their special properties, such as easy solid separation, nanoscale size, high surface-to-volume ratio, high biocompatibility, magnetic separation, and reusability. These laboratory-scale approaches can be further developed on a larger scale. Current strategies in water treatment methods should be focused on sustainable environmental protection and conservation, economic acceptability, and higher efficiency.

A review on pharmaceuticals removal by magnetic adsorbents has been recently published by Tatarchuk et al. [170], who draw attention to the uncontrolled release of pharmaceuticals into aquatic systems; this represents a real threat for living beings and needs urgent and efficient methods of remediation. Adsorption is one of the best and most cost-effective methods for removing contaminants from water. Magnetic adsorbents receive special attention due to their remarkable magnetic properties, chemical stability, high surface reactivity during functionalization, and modification capacity. This study is focused on two pharmaceutical categories: antibiotics (tetracycline, levofloxacin and ciprofloxacin) and non-steroidal anti-inflammatory drugs (ibuprofen, diclofenac, etc.). These drugs mainly adsorb through π → π interactions, hydrogen bonds, surface complexation, ion exchange, etc. Experimental data have shown that adsorption capacity depends on the initial concentration of the pollutant, the solution pH, and the temperature.

Costa et al. [171] evaluated the removal of NSAIDs from water with activated carbons synthetized from waste murumuru. Actually, the adsorption of ibuprofen and naproxen was evaluated using activated carbon doped with ZnCl_2_. The effect of the adsorbent dose, the pH, and the contact time have been evaluated by batch adsorption experiments. The adsorption mechanisms of the process have been established by kinetics and thermodynamics studies. The kinetics followed the pseudo-second-order model, having diffusion as the rate-determining step. The adsorption equilibrium was well fitted according to the Freundlich isotherm. The results obtained demonstrated that activated carbon can be used as an efficient adsorbent for the removal of ibuprofen and naproxen from wastewater.

Experiments at the pilot scale have been performed by Gallardo-Altamirano et al. [172]; they showed that the environmental/operating variables that are specific to the A^2^O combined system, including anaerobic/anoxic/aerobic zones, significantly influenced both the removal efficiency of anti-inflammatory/analgesic pharmaceuticals and the occurrence and abundance of total bacteria and fungi. However, no significant changes in the stability or the resilience of this combined system were observed. The improved removal efficiencies of these analysed pharmaceuticals—ibuprofen, naproxen, ketoprofen, acetaminophen, etc.—are correlated with higher organic content in the influent water, higher concentrations of mixed liquor suspended solids, lower operating temperatures, and lower food–microorganism ratios.

An overview of the agro-industrial residues that have been used as biosorbents for anti-inflammatory contaminant removal from aqueous matrices has been presented by Michelon et al. [173]. By thermal treatment, such residual materials have been converted into highly efficient adsorbents like biochar and activated carbon. However, in order to obtain proper parameters for plant design at a large scale, extended research is still needed.

Moreover, Kaur et al. [174] reported on the degradation of NSAIDs by heterogeneous photocatalysis. Their study provides information on the occurrence of pharmaceutical contaminants and the advances of their treatment techniques. The main operational variables involved in the cleaning process during the degradation of pharmaceuticals, like kinetics and removal efficiency, are carefully monitored. The classical treatment methods that are currently used are not effective enough to be used in the treatment of pharmaceutical wastewater; as a result, there is a need to develop alternative methods. Adsorption is an alternative technique for drug removal, but it does not work for all medicines. Even though activated carbon has high capacity to adsorb pharmaceutical pollutants, its separation from water is rather difficult to achieve. Among the possible remediation alternatives, heterogeneous photocatalysis seems to meet the requirements for NSAIDs removal. In this context, the optimization of operational parameters is also addressed—such as the initial drug concentration, catalyst loading, the pH of the solution—improve the efficiency of the processes. However, there are only a few studies that have directly addressed the identification of the intermediary compounds that form during the degradation reaction. This is why it is necessary to determine the photocatalytic degradation mechanism of drugs during photocatalysis treatment using visible and solar light active catalysts—only a few studies have been proposed on this topic so far. In order to enhancing the solar efficiency of wide-band-gap semiconductors under solar irradiation, it is necessary to modify the composition of nanomaterials, including adequate chromophores, to ensure the enhanced absorption of visible light.

The removal of NSAIDs from water by zeolite-like composites has been studied by Smiljanić et al. [140]. The composites contained clinoptilolite and phillipsite and two cationic surfactants, cetylpyridinium chloride and Arquad 2HT-75. These materials have been tested for the adsorption of ibuprofen and naproxen, and the interference of inorganic anions has been demonstrated.

Recent updates on NSAIDs occurrence, fate, hazards, and removal technologies have been presented by Lin et al. [167]. In their paper, a comparative analysis of the benefits and drawbacks of different removal technologies, including advanced oxidation processes, biodegradation, and adsorption, is presented, and strategies for future development are proposed.

An efficient method for the removal of the recalcitrant contaminant metamizole from drinking water by using a perovskite supported on waste polyethylene nanoparticles has been reported by Valadez-Renteria et al. [175]. Raman analysis and X-ray photo-electron spectroscopy indicated the formation of oxygen vacancy defects that counteract the electron–hole recombination, having the enhancement of contaminant degradation as a final result.

A critical review on the recent advances in pharmaceutical removal from wastewater is presented by Kumar at al. [176], who have been focused on using microbial fuel cells in bio-filtration and bio-nanotechnology procedures. Rapid population growth, industrial expansion, and intensive agricultural practices have created a pressing need for safe and clean water resources. Accelerated urbanization and modernization have led to an exponential increase in the release of pharmaceuticals and personal care product contaminants into aquatic environments and soils. As a result, the conventional methods that are used in the management of pharmaceutical waste cannot achieve the complete degradation of these contaminants; thus, a significant proportion of such wastes remain in the environment. Future evolutions in the study of the removal of pharmaceutical residues from water and soil must improve our engineering methods and develop novel bioremediation techniques that are economically feasible and eco-friendly. While previous research has mainly focused on finding adequate techniques for increasing the removal efficiency of pharmaceutical pollutants from the environment, new microbial technologies are integrated with renewable energy facilities, like wind and the captors of solar irradiation. In this context, innovative environmentally friendly pharmaceutical treatment methods should be developed in the near future, in accordance with the recent progress that has been made in this emerging field.

Another review study has been devoted to the application of semiconductor heterojunctions in the photocatalytic removal of sulphonamide contaminants [177]. The perpetual proliferation of antibiotics in all environmental factors has resulted in the unprecedent spreading of multi-drug-resistant bacteria, which has increased the need for finding adequate solutions for their degradation and removal. Sulphonamides are among the first systematically used antibiotics for the treatment of bacterial and protozoan infections. These contaminants are poorly biodegradable, resulting in the increased antibiotic resistance of bacteria in aqueous ecosystems. Among the most efficient remediation procedures, the following techniques are presented, compared, and analysed: advanced oxidation, photocatalysis, and semiconductor heterojunctions.

França et al. [178] demonstrated that montmorillonite-based adsorbents are versatile materials for drug adsorption. The persistence of pharmaceutical contaminants containing antibiotics, hormones, anti-inflammatory agents, and other organic pollutants present in aquatic systems has strongly increased the request for new remediation techniques. Such an alternative technique is adsorption, using widespread, cost-effective, and natural clay-mineral-type materials. One of the most widely used minerals is montmorillonite, which is characterized by valuable physicochemical properties: adsorption, cation exchange capacity, pollutant intercalation, etc. Moreover, the inclusion of some organic and/or inorganic compounds in the functionalization reaction may change the hydrophobic and/or hydrophilic character of the montmorillonite surface. The formation of new active sites on the montmorillonite surface may enhance its affinity to some chemical species and confer higher selectivity for adsorptive processes. It is worth emphasising that most studies on drug adsorption that use raw or modified montmorillonite have been carried out in batches or columns under ideal conditions. Thus, both raw and organophilic montmorillonites interact with cationic drugs, and their performances may be improved through different chemical modifications, so further research is required. This is particularly important, since topics like the regeneration of adsorbents, biocompatibility, and ecotoxicity are still missing from the literature. Despite the limitations of each montmorillonite adsorbent, they are able to complement each other, as they contribute to various extents to drug adsorption, as a result of the extant differences among the various sites. In this way, adsorption may be applied for a wide variety of pharmaceutical contaminants. Among them, cationic and zwitterionic drugs are the best targets for both natural and modified adsorbents, while adsorption occurs mainly by ion exchange mechanisms. However, there will be long time before montmorillonite-based adsorbents might be currently used at large-scale applications for the removal of pharmaceutical drugs.

Even though there are many studies focusing on understanding physicochemical interactions at interfaces, an important gap exists between the laboratory step and large-scale column adsorption plants. One may conclude that adsorption measurements conducted in batch and column experiments demonstrate that montmorillonite-based adsorbents are versatile, non-toxic, and promising materials for drug adsorption and their removal from environment.

A selective list of PhPs (with their classes and chemical and structural formulas) for which removal methods are presented and discussed in Section 3.1, Section 3.2 and Section 3.3 are displayed in Table A3 (Appendix A.3), with corresponding cited references.

A list of most representative PhP classes, for which waste removal is discussed in Section 3, are displayed in Table A4 (Appendix A.4), with corresponding cited references.

The removal methods for several PhPs or classes of PhPs are shown in Table A5 (Appendix A.5).

### 3.4. Remediation Potential and Strategies

Papers such as [179] aim to propose viable solutions to this global, thorny problem by presenting a general picture of worldwide PhP contamination and by offering regulatory suggestions and educational perspectives. The need for generalized, national, and international regulations is systematically sustained in [180]; here, the international regulatory regime for pharmaceutical pollution is discussed, and international treatises within the Strategic Approach to International Chemicals Management are comparatively analysed in order to generalize a limited scope into a holistic approach. Moreover, recent cooperation efforts and policy initiatives at the global level have been analysed, and new legislations have been proposed.

Policy and regulatory frameworks for controlling pharmaceutical pollution, which must be established based on adequate risk assessment data (currently insufficiently developed), are the remediation approaches reported in paper [37]. A proposal on complex environmental regulation is detailed by Narvaez and Jimenez [102] as a solution for preventing the effects and diminishing the risks of PhP contamination in the environment. A comprehensive discussion on policy implications and future directions for managing pharmaceuticals in the environment was performed by Ashiwaju et al. [105]; here, current regulations and their effectiveness were analysed, together with possible future research trends. It was also specified that a substantial research effort is needed to complete our current understanding and to obtain the database that is required for policy development to be viable.

Regarding solutions that are based on the improvement of the available treatment methods, the use of biological instruments or combinations of biological treatments with conventional wastewater cleaning approaches was proposed by Samal et al. [101] as an appropriate way to mitigate the global spread of PhP contaminants. Other treatment solutions are sustained by Kayode-Afolayan et al. [100], where the utilization of microalgal biomass for waste degradation and water rehabilitation is sustained. The development of bioremediation technologies is largely debated by Ortúzar et al. [107], where a huge number of studies is involved to prove their efficiencies and applicability on certain classes of medicines. The remediation potential of algae is extensively discussed in [181], where a great quantity of thematic studies were analysed in order to demonstrate the advantages, challenges, and future perspectives of phycoremediation techniques for the elimination of PhP contamination. Solutions for the management of PhP pollution, and suggested approaches for choosing appropriate methods for the phytoremediation of pharmaceuticals, are proposed in [103]; AOPs are also discussed here. Taking their efficacy into account, the benefits and limitations of the two methods are compared. In [182], several biological, nanotechnological, and bionanotechnological methods for PhC removal were analysed and compared; here, general conclusions, together with adequate proposals for remediation, are presented.

An ample strategy is proposed by Caban and Stepnowski [183]: after detailed analysis of the production, trajectories, and fates of PhCs, many possible prevention and depollution solutions are presented. Moreover, a comprehensive scheme is provided to clarify the possible approaches for these solutions, and to impress upon the readers’ consciousness the great alarm that must be raised if we are to solve this global problem.

As has been discussed, it is obvious that pharmaceutical products are essential for the health of humans and animals. However, the increases in production and consumption (annually increasing by 6.5% [184]) have become worrisome, with evidence for the presence of PhP pollutants in aquatic systems, and in natural and drinkable water. The technical literature and specialized reports from researchers in different countries indicate high concentrations of PhP compounds in surface and ground water. Although many scientific publications have been published, the acute and chronic effects of PhP pollutants on flora, fauna, and humans are not yet well clarified. The maximum admitted limits for PhC concentrations in water/the environment are not stipulated or enforced by regulations. Only in Australia have some regulations for underground water been established [185].

Most treatment plants are not specifically designed to remove PhP pollutants from wastewater; the pharmaceutical substances used in agriculture and aquaculture are often directly discharged into natural aquatic systems. Of the pharmaceuticals that are orally taken by humans or animals, 40–90% are discharged into public sewage. The conventional wastewater cleaning plants that are equipped with primary and secondary levels do not, generally, remove pharmaceutical micropollutants. Treatment plants where disinfection with free residual chlorine is applied can eliminate about 50% of PhP micropollutants. Advanced treatments (adsorption on activated carbon, micro-, ultra-, and nanofiltration, reverse osmosis, advanced oxidation, and ozonation) can remove PhCs from wastewater or from water destined for human consumption. Nanofiltration and reverse osmosis can remove them almost completely. For instance, by applying the reverse osmosis, more than 99% of big PhC molecules can be eliminated.

### 3.5. Technical and Economic Aspects

An important problem that must be resolved when concrete achievements can be implemented is related to the technical and economic aspects of this issue—i.e., costs in terms of money, energy, and other resources.

In the case of ozonation, electrical energy is necessary for O_3_ generation (highly dose-dependent) and, for major consumables and resources, electricity is required for the ozone generator, the oxygen/air feed, the off-gas management, the potential formation of byproducts, and the post-polishing (GAC/sand) stage. For GAC (granular) polishing (depending on bed life, throughput, regeneration), there are low operational energy costs; the same is true for GAC production and replacement/regeneration (kg GAC per 1000 m^3^). Regarding PAC (dose to mixed liquor, chemical cost, and handling), minimal electricity is required (dosing pumps); a typical PAC dose is 10–50 mg/L (higher doses for refractory MPs). In the case of UV-based AOPs (UV/H_2_O_2_, UV/Cl), there are costs for energy and H_2_O_2_; the energy consumption is highly dependent on UV dose, water UVT, and the electricity supplied to the UV lamps; moreover, hydrogen peroxide (or chlorine), lamp replacement, and prefiltration are all necessary. For nanofiltration or reverse osmosis membranes, high-pressure pumping energy, membrane replacement, concentrate disposal, and pretreatment are all necessary factors to consider.

A cost-effectiveness analysis can be taken into account as an important part of studies on the choice of methods; operational costs are crucial for practical applications in real effluents. It is necessary to consider all expenditures within the economic plan to ensure that the proposed technologies are truly cost-effective and competitive. Works establishing criteria and standardized methods for guiding research in the evaluation and optimization of costs are of crucial importance [108].

## 4. Conclusions, Proposals, and Recommendations

This paper offers an extended overview on pharmaceutical waste pollution, accounting for the occurrence of pharmaceutical contaminants, their pollution sources, their trajectories and concentrations, procedures for monitoring them, existing techniques for their removal, their impacts on environment, and their risks. Methods for removing pharmaceutical pollutants, as well as their remediation potential and strategies for this, have been summarised from a multitude of works. All the included research materials have been collected and organized in order to achieve a general overview and provide a clear understanding of this huge, worldwide problem and to offer remediation solutions and actionable strategies.

The rehabilitation of wastewater cleaning and treatment plants—built through the completion of extant technologies—as an exclusive measure, cannot solve the PhCs problem. Some limitations remain regarding the efficiency of the removal of PhCs: high investment and utilisation costs and high energy consumption. Moreover, rehabilitation does not solve the problem of diffuse PhP pollution (e.g., it does not address the contamination of agriculture and aquaculture).

In the rehabilitation of existing wastewater cleaning and/or treatment plants and in the design of new ones, it is particularly important to consider the realization of complex mathematical models that are able to characterize the flow of liquids or solids through membranes and the phenomenon of the filtration of different PhP pollutants.

The increasing consumption of medicines, which multiplies from year to year, and their growing impact on the environment and human health necessitate the intensification of research in this area.

The presented investigation into the worldwide situation regarding the contamination of aquatic systems with PhCs led to the above-formulated conclusions. Having examined the findings and assertions in the literature, it is important to highlight now some recommendations/proposals:

- It is necessary to draw up an inventory of the PhPs that are used for human and animal purposes which are contaminating water systems across different countries.
- We must include PhC monitoring in programs for water quality control from natural water sources, wastewater, and drinkable water based on research which must be performed a priori to monitor some PhCs. However, such research must not distract from the attention given to the control of existent parameters and indicators.
- It is very important to establish a methodology for sampling and determining PhCs from water; special attention should be paid to the standardization of the accepted limits for PhC concentrations in natural water, wastewater, and drinkable water.
- It is very important to perform appropriate research to establish the effects of the presence of PhP micropollutants in drinkable water and aquatic systems in general on humans and on flora and fauna, respectively.
- The initiation/continuation of research to establish appropriate rehabilitation solutions for existent wastewater cleaning and treatment plants for the removal of micropollutants (including PhCs) from water.

An urgent priority is the initiation of research aiming to identify solutions for water supply in regions without centralized systems. The specific conditions of many disadvantaged areas pose significant challenges in ensuring safe water provision for public health.

## Data Availability

No new data were created or analyzed in this study. Data sharing is not applicable to this article.

## Abbreviations

The following abbreviations are used in this manuscript:

PhPs	pharmaceutical products
POPs	persistent organic pollutants
DDT	dichlorodiphenyltrichloroethane
PCBs	polychlorinated biphenyls
HCH	hexachlorocyclohexane
PhCs	pharmaceutical contaminants
CFC	chlorofluorocarbon
PAHs	polyaromatic hydrocarbons
OECD	Organization for Economic Co-operation and Development
HC	human consumption
VC	veterinary consumption
NSAIDs	nonsteroidal anti-inflammatory drugs
HDCs	human drug consumers
ADCs	animal drug consumers
DWC	drinking water consumer
PhPHs	pharmaceutical products for human use
PhPVs	pharmaceutical products for veterinary use
MSTs	multivariate statistical techniques
PCA	principal component analysis
MANOVA	multivariate analysis of variance
ANOVA	analysis of variance
AOPs	advanced oxidation processes
NTs	nanotubes
COD	chemical oxygen demand
PPCPs	pharmaceutical and personal care products
EAOPs	electrochemical advanced oxidation processes
CTAB	cethyltrimethyl ammonium bromide

## Appendix A

### Appendix A.1

Selective PhCs or classes of PhPs and their concentrations—found in surface water, underground water, on estuaries, or in seawater in different countries—for which we found measured values are displayed in Table A1, together with cited references. This table only represents a sample. An inventory should be completed using global information.

**Table A1:** Selective list of some pharmaceutical products or classes of pharmaceutical products, together with their detected concentrations, from a few countries and ecosystems. Values were found in the cited literature.

PhPs/Class and Application/Usage	Concentration Level Detected, ng/L	Ecosystem	Country/Citation
Azithromycin/Antibiotic/HC	2.6–166.5	Surface	China/[2]
Ciprofloxacin/Antibiotic/HC	1.5–2.4	Surface	Sweden/[2]
Clarithromycin/Antibiotic/HC	123	Surface	China/[2]
Chloramphenicol/Antibiotic/HC	0.0002–0.016	Surface	Sweden/[2]
Erythromycin/Antibiotic/HC and VC	80	Surface	UK/[1]
	6.46	Surface	Bangladesh/[2]
	22.3	Surface	China/[2]
	2.2–54.8	Underground	Taiwan/[2]
Metronidazole/Antibiotic/HC	1.41–156	Surface	China/[2]
Norfloxacin/Antibiotic/HC	4.5–83	Surface	UK/[1]
	2.8–9.3	Underground	Taiwan/[2]
Roxithromycin/Antibiotic/HC	0.6–88.4	Surface	China/[2]
	0.0013	Surface	Sweden/[2]
Sulfamethazine/Antibiotic/HC and VC	3.7	Surface	China/[2]
	4.19	Surface	Bangladesh/[2]
	0.3–28.9	Underground	Taiwan/[2]
Sulfamethoxazole/Antibiotic/HC and VC	0.55	Surface	UK/[1]
	0.44–115.3	Surface	China/[2]
	11.35	Surface	Bangladesh/[2]
	0.1–1820	Underground	Taiwan/[2]
Tetracycline/Antibiotic/HC and VC	1000	Surface	UK/[1]
Trimethoprim/Antibiotic/HC	17.20	Surface	Bangladesh/[2]
	15.6	Surface	China/[2]
	0.33	Surface	Sweden/[2]
	0.0–0.68	Surface	USA/[2]
Aspirin/Analgesics and anti-inflammatories/HC and VC	25–294	Surface	Columbia/[2]
	0.0007	Seawater	Sweden/[2]
	2.5	Underground	Serbia/[2]
Acebutolol/Antihypertensive/HC	0.7	Underground	Taiwan/[2]
Atenolol/Antihypertensive/HC	181	Surface	China/[2]
	3.5–8.5	Surface	Sweden/[2]
	0–14.2	Lake	USA/[2]
	3.6	Underground	Taiwan/[2]
Metoprolol/Beta-blocker/HC	0.46–17.3	Surface	China/[2]
	0.04–21.2	Estuary	China/[2]
	3.5	Underground	Serbia/[2]
	0.04–21.2	Underground	Taiwan/[2]
Propranolol/Beta-blocker/HC	29	Surface	UK/[1]
	0.9	Estuary	China/[2]
	0.0006	Surface	Sweden/[2]
	4.5	Underground	Serbia/[2]
Furosemide/Antihypertensive/HC	4.7–9.4	Surface	Sweden/[2]
Verapamil/Antihypertensive/HC	0.053	Surface	Sweden/[2]
Carbamazepine/Anticonvulsants/HC	8.80	Surface	Bangladesh/[2]
	25.3, 21.3, 0.41–10.3	Surface	China/[2]
	37.4	Estuary	China/[2]
	0.38–0.51	Surface	Sweden/[2]
	3.4	Underground	Serbia/[2]
Diazepam/Antidepressants/HC	24.3	Surface	China/[2]
	6.1	Estuary	China/[2]
Gabapentin/Antiepileptic/HC	4.5	Surface	Sweden/[2]
Cimetidine/Antihistamine/HC	0.36	Surface	Sweden/[2]
Fluoxetine/Antidepressants/HC	5	Surface	UK/[1]
	0.4	Surface	China/[2]
	0.4–4.7	Surface	Sweden/[2]
Simvastatin/Lipid-lowering agent/HC	2.3–16.7	Estuary	China/[2]
Tramadol/Analgesic/HC	3.01	Surface	Sweden/[2]
Diclofenac/NSAIDs/HC and VC	195	Surface	UK/[1]
	20–64	Surface	Austria/[1]
	18–41	Surface	France/[1]
	150–1200	Surface	Germany/[1]
	1.5	Surface	China/[2]
Ibuprofen/NSAIDs/HC and VC	826–5044	Surface	UK/[1]
	23–120	Surface	France/[1]
	70–530	Surface	Grece/[1]
	0.86–115.8	Surface	China/[2]
	0.2	Underground	Serbia/[2]
	7–836.7	Underground	Taiwan/[2]
Ketoprofen/NSAIDs/HC and VC	1.4–54.5	Estuary	China/[2]
	75.3	Surface	Colombia/[2]
	10.3–89	Seawater	Portugal/[2]
Naproxen/NSAIDs/HC and VC	3.5	Surface	China/[2]
	260	Surface	Colombia/[2]
	1178	Seawater	Portugal/[2]

### Appendix A.2

Selective effects drawn as impacts and risks assessment of PhCs or classes of PhPs on environment and human health are displayed in Table A5 together with their cited references. It is notable that the effects caused by drugs on the sampled aquatic area are note yet adequately known or interpreted. It was confirmed that the ecotoxicological models do not completely present the effects of pollutants on the organized aquatic 356 systems; ecotoxicological tests are very important forms of analysis in the description of the destructive effects of active contaminants on organism life.

**Table A2:** Selective list of some pharmaceutical products or classes of pharmaceutical products together with their determined impacts and risk assessments. Details found in the cited literature.

Pharmaceutical Compound/Class	Impact on the Environment/References	Risk Assessment/References
Antibiotics (Inhibiting and toxic compounds)	Antibiotic resistance/[100,186,187] Haematological and hormonal imbalances in fish/[188,189,190,191] Growth of phytoplankton/[101] Great negative effects on marine amphipods & aquatic invertebrates/[101]	Antibiotic resistance/[100,186,187] Marine biodiversity/[69,103,108] Aquatic ecosystems/[43,103,104] Antimicrobial resistance/[105,106] Worldwide effects/[107]
Alcohols, acetone (High content of organic matter)	Oxygen depletion/[100,192,193] Eutrophication (algal bloom)/[194]	Marine biodiversity/[100] Mussels’ digestive glands/[104] Worldwide effects/[107]
Aromatic compounds, chlorinated hydrocarbons (Slowly biodegradable organic compounds)	Disruption of immune responses in aquatic fauna/[100,195,196]	Effects on aquatic and terrestrial wildlife/[105] Potential human exposure/[105] Worldwide effects/[107]
NSAIDs	Great negative effects on marine amphipods & aquatic invertebrates/[101]	Aquatic ecosystems/[103] Worldwide effects/[107]
Beta-blockers	Great negative effects on marine amphipods & aquatic invertebrates/[37]	Biodiversity/[37] Worldwide effects/[107]
Analgesics	Human health and environment/[43]	Ecological health risks/[43] Worldwide effects/[107]

### Appendix A.3

The PhPs for which residues disposal is discussed in Section 3.2 are displayed in Table A3 together with cited references.

**Table A3:** List of most representative pharmaceutical products, their chemical and structural formulae, and reference sources.

Pharmaceutical Product/Class and Therapeutical Application	Chemical Formula and Molecular Mass (g/mol)	Structural Formula	References
Ibuprofen/ NSAIDs	C_13_H_18_O_2_ 206.285		[113,114,121,124,126,140,143,152,153,155,159,164,197]
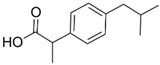
Naproxen/ NSAIDs	C_14_H_14_O_3_ 230.263		[118,120,121,140,144,147,148,166]
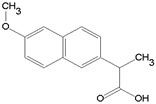
Ketoprofen/ NSAIDs	C_16_H_14_O_3_ 254.285		[111,114,139,144,155,158,166]
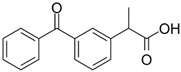
Diclofenac/ NSAIDs	C_14_H_11_C_l2_NO_2_ 296.147		[122,124,125,146,149,150,151,158,162,164]
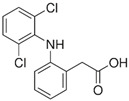
Aspirin/ Analgesic and anti-inflammatory	C_9_H_8_O_4_ 180.159		[162]
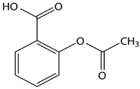
Paracetamol (Acetaminophen)/ Analgesic and anti-inflammatory	C_8_H_9_NO_2_ 151.063		[116]
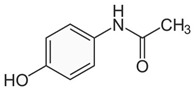
Sulfamethoxazole/ Antibiotic	C_10_H_11_N_3_O_3_S 253.28		[143,198]
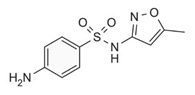
Carbamazepine (Tegretol)/ Anticonvulsant and neuropathic pain	C_15_H_12_N_2_O 236.274		[153,161]
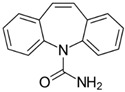

### Appendix A.4

The PhP classes for which waste removal is discussed in Section 3.3 are displayed in Table A4, with corresponding citations from the literature.

**Table A4:** List of most representative classes of pharmaceutical products studied in our paper and their referenced sources.

Class and Therapeutical Application	PhPs	References
NSAIDs	Ibuprofen, Naproxen, Ketoprofen, Diclofenac, Acetaminophen (Paracta-mol), Acetylsalicylic acid (aspirin), Fenoprofen, Mefenamic acid, Metho-carbamol, Chlorzoxazone, Lidocaine hydrochloride, Indomethacin	[40,44,121,128,140,141,145,155,156,157,160,161,167,168,170,171,172,173,174,175,176]
Analgesic PhPs	Propyphenazone, Indomethacin, Codeine, Dexamethasone, Tramadol	[127,157,172,178]
Antibiotics	Amoxicillin, Cefaclor, Cefazolin, Ciprofloxacin, Sulfadimethoxine, Tetracycline, Trimethoprim, Cefotaxime, Chlortetracycline, Oxytetracycline, Levofloxacin, Enrofloxacin, Norfloxacin, Daptomycin, Erythromycin, Streptomycin, Cephalexin, Metronidazole, Sparfloxacin, Cefotaxime, Ceftriaxone, Chloramphenicol, Ampicillin, Enrofloxacin, Marbofloxacin, Penicillin G, Trimethoprim, Clarithromycin, Sulfamethoxazole	[40,44,141,170,178,199]
Hormones/ Endocrine disrupter	17-alpha-ethinylestradiol, Bisphenol A, Nonylphenol	[40,156,178,200]
Proton pump inhibitors	Omeprazole, Ranitidine	[40]
Anticonvulsant PhPs/Neuropathic pain/ Antidepressant	CBZ-Diol, Carbamazepine, Gabapentin, Lamotrigine, Amitriptyline, Diazepam, Doxepin, Imipramine, Oxazepam, Amisulpride, Citalopram, Venlafaxine	[141,157,178,197]
Beta-blocker/ Antihypertensive	Acebutolol, Atenolol, Bezafibrate, Clofibric acid, Gemfibrozil, Losartan, Metoprolol, Propranolol, Hydrochlorothiazide	[141,161,178,201]
Antihistamine/ Antiallergic	Chlorpheniramine, Dexamethasone	[178]
Antifungal	Clotrimazole, Econazole	[40]

### Appendix A.5

Selective PhPs or classes of PhPs for which removal methods are presented and discussed in Section 3.1, Section 3.2 and Section 3.3 are displayed in Table A3 together with cited references.

**Table A5:** Selective list of representative pharmaceutical products or classes of pharmaceutical products together with their removal methods studied in our paper and their referenced sources.

Pharmaceutical Compound/Class	Removal Methods/Citation
NSAIDs	AOPs/[108,110,130,132]
NSAIDs	EAOPs/[128]
NSAIDs	Activated carbon/[156]
Naproxen and Diclofenac	Advanced Membranes Technologies/[110,137]
Antibiotics	AOPs/[129,130,132,134]
Antibiotics	Advanced Membranes Technologies/[133]
Diazepam and Carbamazepine	AOPs/[132]
Ketoprofen	AOPs/[111]
Ketoprofen	Ultrafiltration and photocatalysis/[139]
Ibuprofen	UV/Fe(III)/Oxone process/[113]
Ibuprofen	Photo-Fenton process/[114]
Ibuprofen and naproxen	Zeolite-based filters/[140,141]
Naproxen and ketoprofen	Carbonaceous adsorbents/[144]
Acetaminophen	Electro-Fenton processes/[116]
NSAIDs	Electro-Fenton processes/[121]
Naproxen	Chlorination and biofilm processes/[147]
Naproxen	Photocatalysis process/[120]
Ibuprofen	Photocatalysis process/[114]
Diclofenac	Photo-electrocatalytic processes/[122]
Ibuprofen	Oxidation & sonophotocatalytic degradation/[126]
Analgesics	AOPs/[127]
Disinfectant	AOPs/[127]
Antibiotics	AOPs/[127]
